# Syntheses, Characterization, and Redox Activity of Ferrocene-Containing Titanium Complexes

**DOI:** 10.1021/acsomega.4c04332

**Published:** 2024-06-24

**Authors:** Kevin Schwitalla, Justin Klimek, Tobias Greven, Marc Schmidtmann, Rüdiger Beckhaus

**Affiliations:** Institut für Chemie, Carl von Ossietzky Universität Oldenburg, D-26111 Oldenburg, Federal Republic of Germany

## Abstract

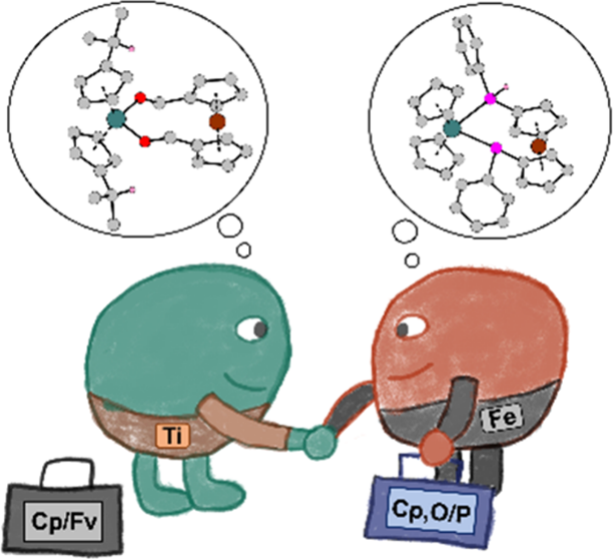

The chemistry of bis(π–η^5^:σ–η^1^-pentafulvene)titanium complexes is characterized by a broad range of E–H activation and Ti–C functionalization reactions, whereas ferrocene derivatives are easily accessible and redox-active compounds. The reaction of ferrocenealdehyde and -ketones with bis(π–η^5^:σ–η^1^-pentafulvene)titanium complexes result in the formation of bimetallic complexes via insertion of the C=O double bond of the aldehyde/ketone into the Ti–C_exo_ bond of the pentafulvene moiety. The reaction of bis(π–η^5^:σ–η^1^-pentafulvene)titanium complexes with ferrocenyl alcohols leads to alcoholate complexes via deprotonation of the OH group by the pentafulvene ligand. Because of the one remaining pentafulvene unit, further functionalization of the complexes is possible. In this work, we proceeded with 1,1′-bifunctionalized ferrocene derivatives for intramolecular follow-up reactions. 1,1′-Ferrocenedimethanol reacts with bis(π–η^5^:σ–η^1^-pentafulvene)titanium complexes in a double O–H deprotonation reaction to yield the dialcoholate complex. 1,1′-bis(phenylphosphine)ferrocene reacts differently as the double P–H deprotonation reaction results in the formation of a P–P linked phosphine. Therefore, we studied the reactivity of 1,1′-bis(phenylphosphine)ferrocene toward Rosenthal’s reagent. As Rosenthal’s reagent is regarded as a masked titanocene(II) species, it undergoes redox reactions toward H-acidic substrates, forming a paramagnetic Ti(III) complex.

## Introduction

The synthesis and applications of heterobimetallic complexes are an ever-present topic in inorganic chemistry.^1−4^ Of particular interest is the incorporation of ferrocene derivatives into other metal complex frameworks.^5−7^ Since the discovery of ferrocene and its formerly unprecedented sandwich-structure,^8,9^ many ferrocene derivatives have been reported.^10−13^ Because of the manifold derivatization options of the ferrocene framework, there is a huge number of polymetallic complexes containing ferrocenes which are linked with different bridges (C;^14−17^ N;^18−22^ O;^23−25^ P;^26^ S;^27,28^ Se;^29,30^ N,O^28,31,32^). In the context of ferrocene-based titanium complexes, several studies have focused on clusters,^33−36^ electron-deficient catalysts^19,37^ and redox-active compounds.^5,38−40^ Because the chemistry of bis(π–η^5^:σ–η^1^-pentafulvene)titanium complexes is characterized by a broad range of E–H activation and Ti–C functionalization reactions,^41^ they are excellent precursors for the generation of bimetallic Ti/Fe complexes.^42,43^ As we reported earlier,^44^ both monofunctional and bifunctional transformations are possible depending on the substrate. Herein, we report novel bimetallic Ti/Fe complexes based on ferrocene aldehydes, ketones, alcohols and 1,1′-bis(phenylphosphine)ferrocene. Although there are several examples of ferrocene-containing titanium alcoholate complexes, we present the first ferrocene-based titanium phosphido complex. The complexes were characterized using NMR spectroscopy, single-crystal X-ray diffraction (SCXRD), computational studies, cyclic voltammetry (CV) and electron paramagnetic resonance (EPR) spectroscopy.

## Results and Discussion

**Scheme 1:**
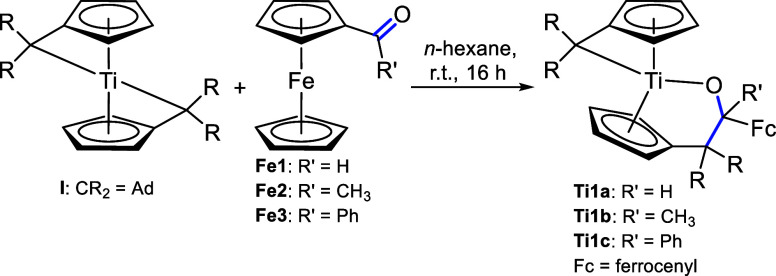
Reaction of Bis(π–η^5^:σ–η^1^-pentafulvene)titanium Complex **I** with Ferrocenealdehyde **Fe1** and Ketones **Fe2**,**3** to the Insertion Products **Ti1a**,**b**,**c**

The reaction of ferrocenealdehyde **Fe1** and ferroceneketones **Fe2** and **Fe3** with bis(π–η^5^:σ–η^1^-pentafulvene)titanium complex **I** results in the formation of insertion products **Ti1a**,**b**,**c** (Scheme 1). The complexes were formed via the insertion of the C=O double bond of the aldehyde or ketones into the Ti–C_exo_ bond, thus forming alcoholate ligands.

**Figure 1:**
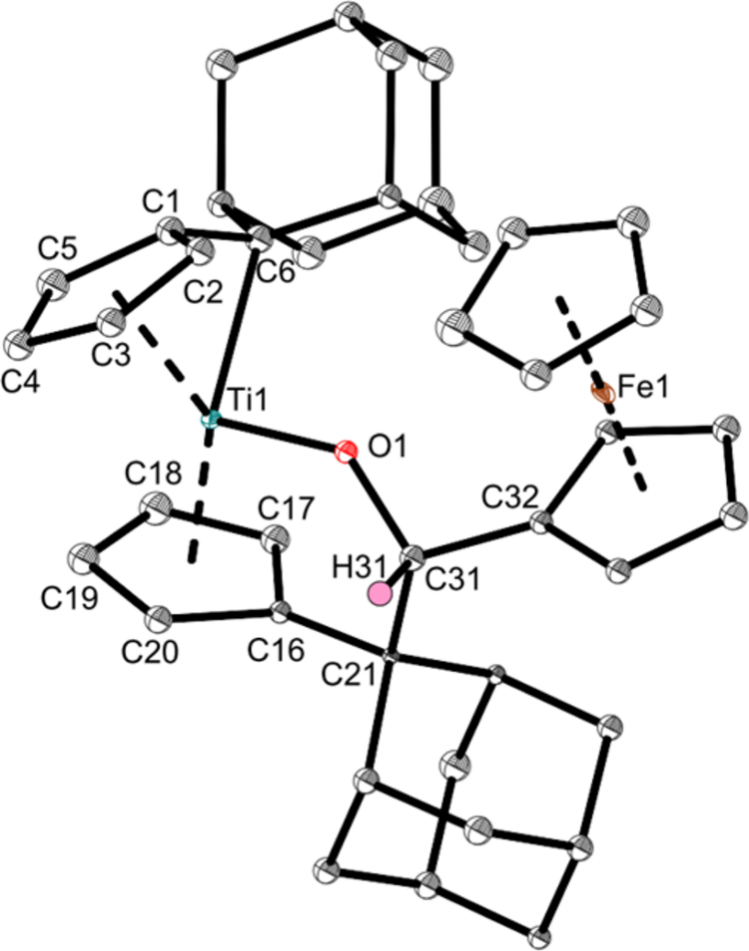
Molecular structure of complex **Ti1a**. Displacement ellipsoids are drawn at the 50% probability level. Redundant H atoms and solvent molecules have been omitted for clarity. Selected bond lengths (Å) and angles (deg): Ti1–O1 1.859(5), Ti1–C6 2.361(7), O1–C31 1.420(8), C31–C32 1.512(9), C31–C21 1.621(9), C1–C6 1.443(10), Ct1–Ti–Ct2 139.0, Ti1–C6–C1 63.6(4), O1–Ti1–C6 104.6(2), C31–C21–C16 104.2(5), Ti1–O1–C31 133.0(4) (Ct1 = centroid of C1–C5; Ct2 = centroid of C16–C20).

The products were characterized via NMR spectroscopy (**SI**, Figures S1–S6) and additionally, the structure of **Ti1a** was determined by single-crystal X-ray diffraction (Figure 1). The ^1^H NMR spectra reveal two sets of signals per complex due to the asymmetric Ti center and the former prochiral aldehyde/ketone, which become chiral after the insertion reaction, forming two diastereomers. One diastereomer is favored in each complex, revealing product ratios of 1:0.4 (**Ti1a**), 1:0.2 (**Ti1b**) and 1:0.25 (**Ti1c**). The eight different signals of the Cp-protons of the main diastereomers indicate an asymmetry between both Cp-rings and therefore shows that the reaction occurred only at one of the pentafulvene units. The ^1^H NMR chemical shift of the former aldehyde signal of **Ti1a** at 5.76 ppm indicates that the insertion reaction occurred because the signal is shielded more strongly in comparison with the respective ferrocenealdehyde (9.93 ppm).^45^ In addition, the ^13^C NMR chemical shifts of the former carbonyl carbon atoms now correspond to aliphatic carbon atoms (**Ti1a**: 54.8 ppm, **Ti1b**: 56.2 ppm, **Ti1c**: 61.0 ppm) compared with the precursors (193.6,^45^ 202.2,^46^ 199.3 ppm^46^).

We abstained from a detailed discussion of bond length, etc. for **Ti1a** due to poor data quality of the structure.

**Scheme 2:**
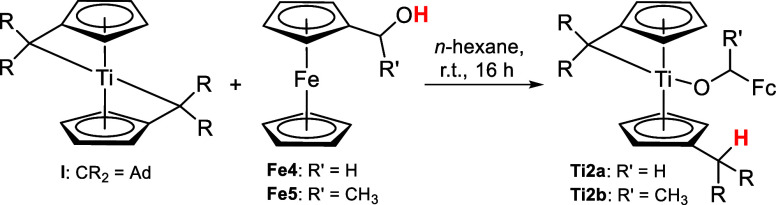
Reaction of Bis(π–η^5^:σ–η^1^-pentafulvene)titanium Complex I with Ferrocenyl Methanol **Fe4** and 1-Ferrocenyl Ethanol **Fe5** to the Alcoholate Complexes **Ti2a**,**b**

The reactions of ferrocenyl methanol **Fe4** and 1-ferrocenyl ethanol **Fe5** with the bis(π–η^5^:σ–η^1^-pentafulvene)titanium complex **I** result in the formation of the alcoholate complexes **Ti2a**,**b** via deprotonation of the OH group by the pentafulvene ligand (Scheme 2).

**Figure 2:**
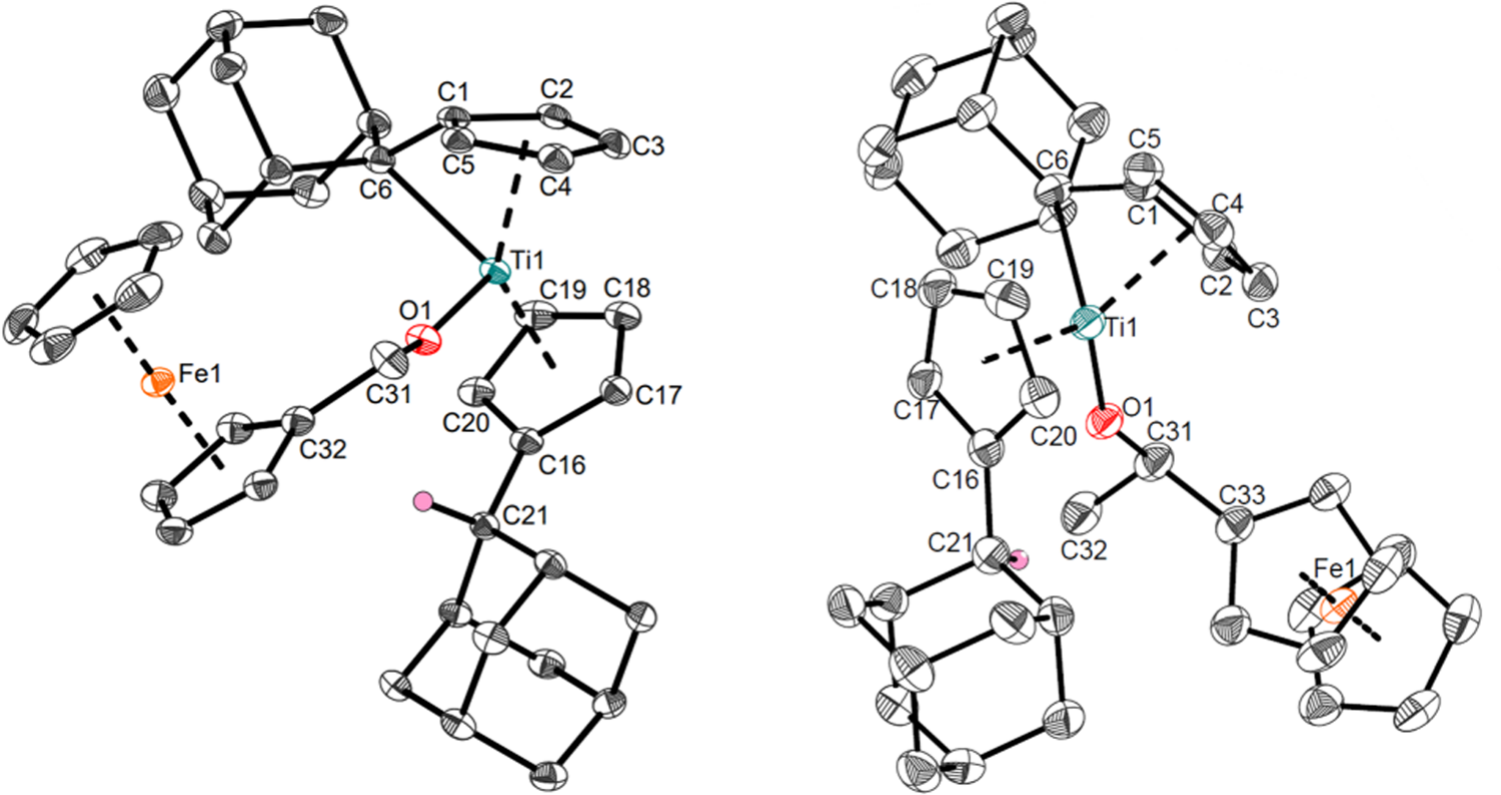
Molecular structures of complexes **Ti2a** (left) and **Ti2b** (right). Displacement ellipsoids are drawn at the 50% probability level. Redundant H atoms have been omitted for clarity. Selected bond lengths (Å) and angles (deg): **Ti2a**, Ti1–O1 1.8362(11), Ti1–C6 2.3761(17), O1–C31 1.3949(19), C31–C32 1.511(2), C16–C21 1.513(2), C1–C6 1.445(2), Ti1–C6–C1 36.76(6), O1–Ti1–C6 102.89(59), C31–O1–Ti1 154.21(10), Ct1–Ti–Ct2 135.9; **Ti2b**, Ti1–O1 1.8536(14), Ti1–C6 2.411(2), O1–C31 1.421(3), C31–C33 1.506(3), C16–C21 1.514(3), C1–C6 1.437(3), Ti1–C6–C1 36.10(8), O1–Ti1–C6 99.26(7), O1–Ti1–C31 150.88(14), Ct1–Ti–Ct2 133.3 (Ct1 = centroid of C1–C5; Ct2 = centroid of C16–C20).

While two diastereomers are formed in **Ti2b** (ratio of 1:0.8) due to the asymmetric Ti center and the racemic **Fe5**, there is only one set of signals for **Ti2a** as **Fe4** is achiral. The eight different signals of the Cp-protons in the ^1^H NMR spectra (SI, Figures S7 and S9) indicate that deprotonation of one equivalent of the alcohols occurred due to the asymmetry of the Cp rings. Both complexes were additionally characterized by single-crystal X-ray diffraction (Figure 2).

The molecular structures of the alcoholate complexes **Ti2a** and **Ti2b** reveal Ti–O bonds (1.8362(11), 1.8536(14) Å) with increased double bond character according to the sum of covalent radii (∑r_cov_(Ti–O) = 1.99 Å,^47^ ∑r_cov_(Ti=O) = 1.74 Å^48^), which is typical for titanium alcoholate complexes (1.847 Å)^49^ and in accordance with other ferrocene-containing titanium alcoholate complexes (1.849(4),^50^ 1.830(2) Å^51^). The Ti–C bonds of the remaining pentafulvene units (2.3761(17), 2.411(2) Å) are similar to the bis(π–η^5^:σ–η^1^-pentafulvene)titanium complex **I** (2.341(2), 2.363(2) Å),^52^ indicating similar reactivity toward other substrates.

**Scheme 3:**
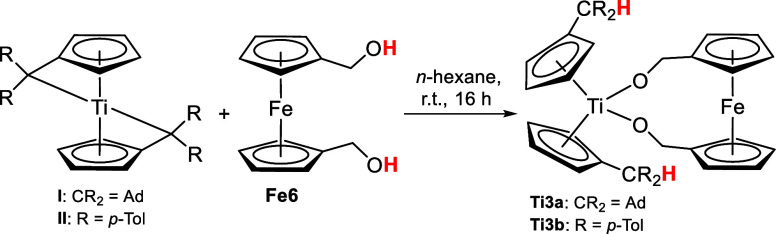
Reaction of Bis(π–η^5^:σ–η^1^-pentafulvene)titanium Complexes **I** and **II** with 1,1′-Ferrocenedimethanol **Fe6** to the Dialcoholate Complexes **Ti3a**,**b**

As the bis(π–η^5^:σ–η^1^-pentafulvene)titanium complexes bear two pentafulvenes, we studied their reactivity toward bifunctional ferrocene-based ligands. 1,1′-Ferrocenedimethanol **Fe6** reacts with the bis(π–η^5^:σ–η^1^-pentafulvene)titanium complexes **I** and **II** in a double deprotonation reaction of the two OH groups to yield the dialcoholate complexes **Ti3a** and **Ti3b** (Scheme 3).

**Figure 3:**
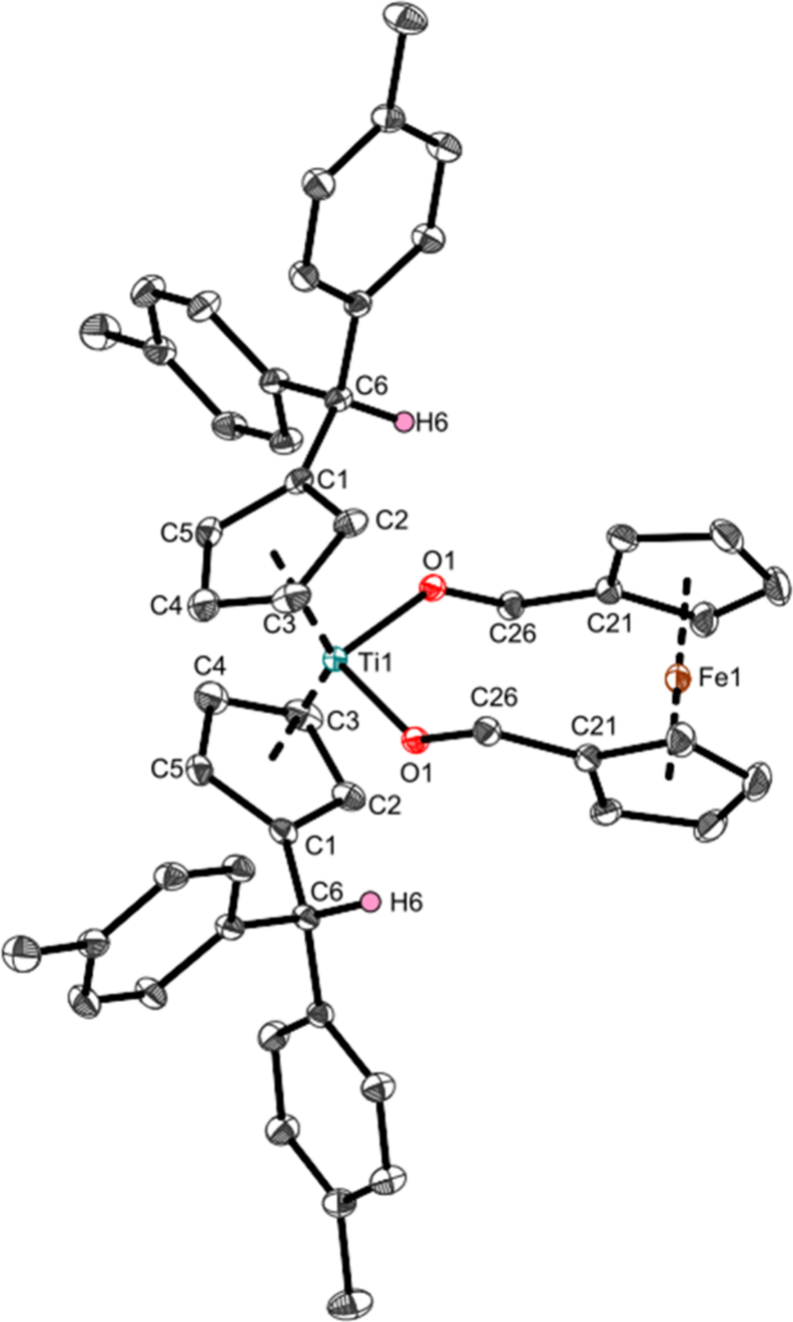
Molecular structure of complex **Ti3b**. Displacement ellipsoids are drawn at the 50% probability level. Redundant H atoms have been omitted for clarity. Selected bond lengths (Å) and angles (deg): Ti1–O1 1.8707(5), O1–C26 1.3980(7), C21–C26 1.5028(9), O1–T1–O1 99.36(3), Ti1–O1–C26 138.78(4), O1–C26–C21 110.86(5), Ct–Ti–Ct 131.1 (Ct = Centroid of C1–C5).

The ^1^H NMR spectra (SI, Figures S11 and S13) indicate that double deprotonation reactions occurred because of the reduced amount of signals. Two signals correspond to the titanium Cp protons, two signals to the ferrocene Cp protons and one signal to the methylene groups, displaying high symmetry. In addition, the molecular structure of the dialcoholate complex **Ti3b** was revealed by single-crystal X-ray diffraction (Figure 3).

The Ti–O bonds (1.8707(5) Å) of **Ti3b** are slightly longer than those of **Ti1a** (1.859(5) Å) and **Ti2a**,**b** (1.8362(11), 1.8536(14) Å) but still contain increased double bond character.

**Figure 4:**
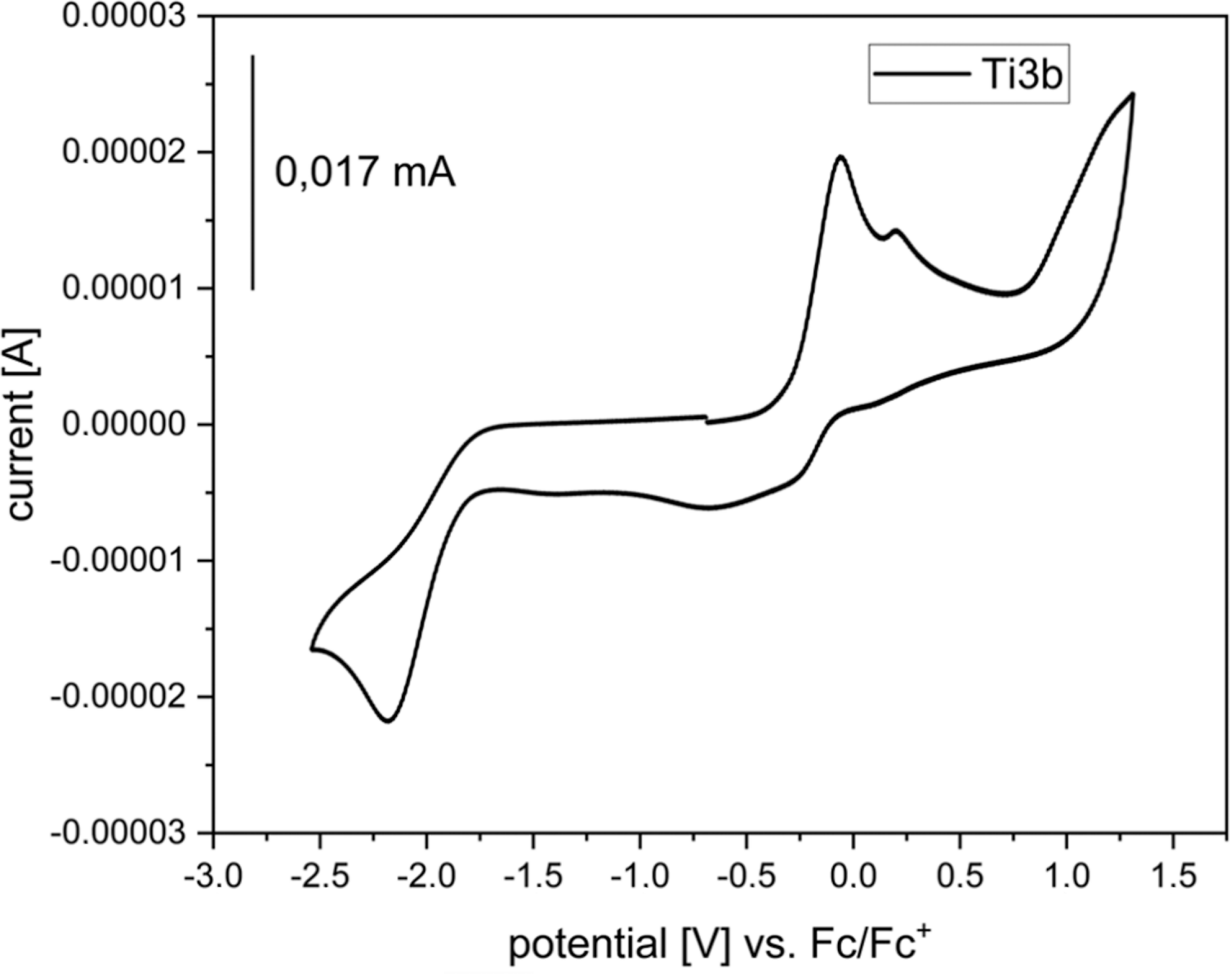
Cyclic voltammogram of complex **Ti3b**. Measurements were performed in THF at room temperature under an argon atmosphere (supporting electrolyte [*n*-Bu_4_N]PF_6_, 0.1 M, scan rate 0.1 V/s). Potentials were referenced to the ferrocene/ferrocene^+^ redox couple with *E*_1/2_ = 0.00 V.

To determine the redox properties of **Ti3b**, we performed cyclic voltammetric measurements (Figure 4). Oxidation of the ferrocene moiety (Fe(II)/Fe(III) *E*_ox,Fc_ = −0.06 V) and reduction of the titanocene moiety (Ti(IV)/Ti(III) *E*_red,Ti_ = −2.18 V) were observed. Both redox processes are irreversible, and while the Ti(IV)/Ti(III) reduction of titanocene alcoholates is often irreversible,^50^ the Fe(II)/Fe(III) oxidation of **Fe6** is reversible (SI, Figure S22). Therefore, the irreversibility of the oxidation of **Ti3b** was unexpected.

**Figure 5:**
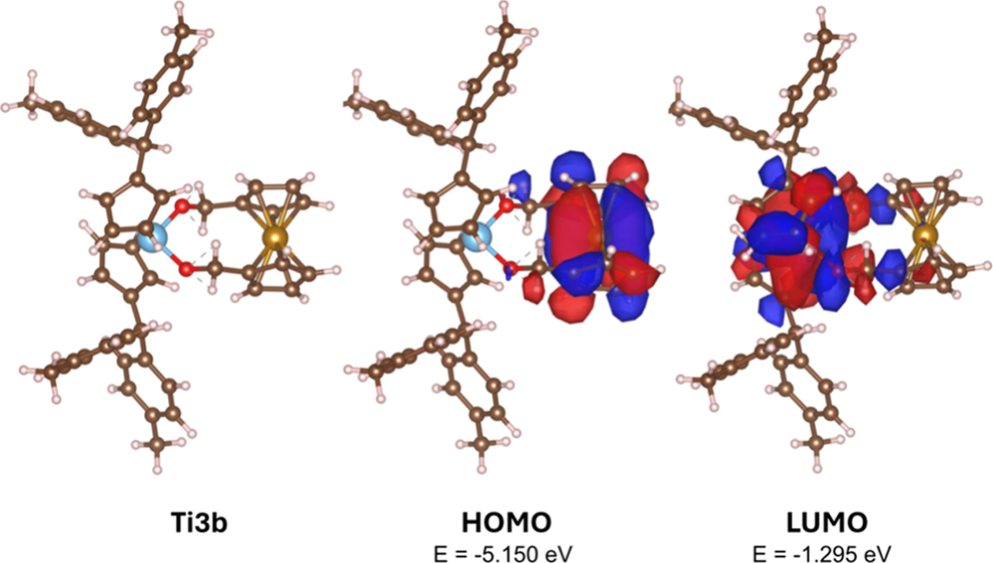
Calculated structure of **Ti3b** without (left) and with the calculated surface diagram of the HOMO (middle) and the LUMO (right)(isodensity value 0.01 at B3LYP/Def2-TZVP).

The frontier orbitals of **Ti3b** (Figure 5) reveal a ferrocene-centered highest occupied molecular orbital (HOMO) and a titanocene-centered lowest unoccupied molecular orbital (LUMO) with no interactions between the metals. The density functional theory (DFT) calculations support the cyclic voltammetry (CV) studies because the ferrocene oxidizes while the titanocene is reduced.

**Scheme 4:**
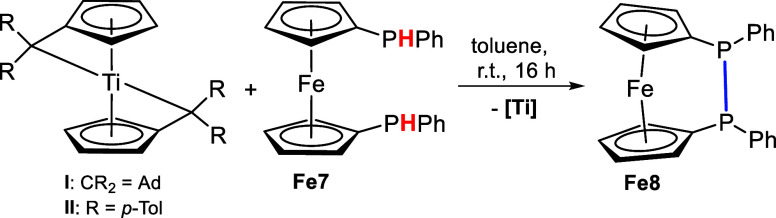
Reaction of Bis(π–η^5^:σ–η^1^-pentafulvene)titanium Complexes **I** and **II** with 1,1′-Bis(phenylphosphine)ferrocene **Fe7** to the P–P-linked Ferrocenephosphine **Fe8**

Furthermore, we used 1,1′-bis(phenylphosphine)ferrocene **Fe7** to demonstrate the reactivity of bis(π–η^5^:σ–η^1^-pentafulvene)titanium complexes toward bidentate phosphine ligands. A double deprotonation reaction of the two PH groups and the formation of a bis(phosphido)titanium complex was expected, however, the reactions resulted in the formation of the P–P-linked ferrocenephosphine **Fe8** (Scheme 4). This is evident from the NMR measurements as the ^31^P{^1^H} NMR spectra (SI, Figures S17 and S20) show only one singlet at 5.3 ppm, corresponding to other P–P-linked ferrocenephosphines (8.7, 20.6 ppm).^53^ The attempted synthesis of **Fe8** was also reported and a chemical shift of 4.6 ppm was found.^53^ In contrast, the precursor **Fe7** shows a doublet at −60.9 ppm, which corresponds to secondary phosphines (SI, Figure S21). The ^1^H NMR spectra (SI, Figures S15 and S18) show the respective signals of **Fe8** along with broad signals of the respective titanium complex species, which were formed as byproducts. The nature of these titanium complex species could not be determined and the product mixture could not be separated.

**Figure 6:**
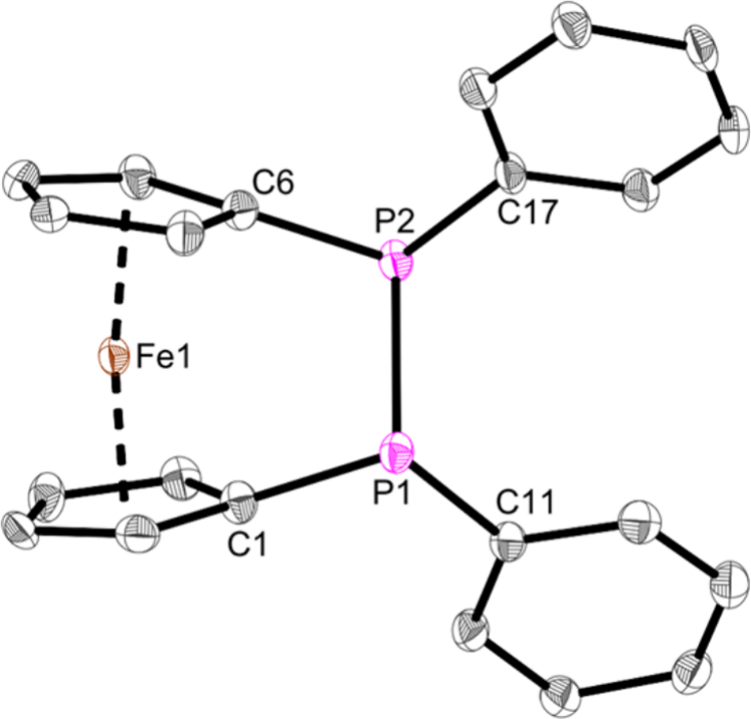
Molecular structure of complex **Fe8**. Displacement ellipsoids are drawn at the 50% probability level. Redundant H atoms have been omitted for clarity. Selected bond lengths (Å) and angles (deg): P1–P2 2.270(2), P1–C1 1.830(6), P2–C6 1.833(7), P1–C11 1.835(6), P2–C17 1.838(6), C1–P1–C11 104.1(3), C6–P2–C17 103.7(3), C1–P1–P2 103.71(7), C6–P2–P1 95.7(2), C11–P1–P2 100.5(2), C17–P2–P1 100.1(2), Ct1–Fe–Ct2 = 171.5 (Ct1 = Centroid of C1–C5; Ct2 = Centroid of C6–C10).

The structure of the P–P linked ferrocenephosphine **Fe8** was revealed by single-crystal X-ray diffraction (Figure 6). The P–P bond (2.270(2) Å) is an elongated single bond and is slightly longer than other P–P-linked phosphines (2.212(1),^54^ 2.217(1),^55^ 2.2345(9) Å^56^). Similar reported P–P linked ferrocenephosphines show the same feature (2.2502(8), 2.3100(7) Å (**Fe8**[Cr(CO)_5_]_2_)).^53^ While the P–C bonds (1.830(6) – 1.838(6) Å) are in accordance with Aryl–P bonds (1.836 Å),^57^ the geometry of the ferrocene is slightly angled (Ct1–Fe–Ct2 = 171.5°). We abstained from a more detailed discussion of bond length, etc. for **Fe8** due to poor data quality of the structure.

**Scheme 5:**
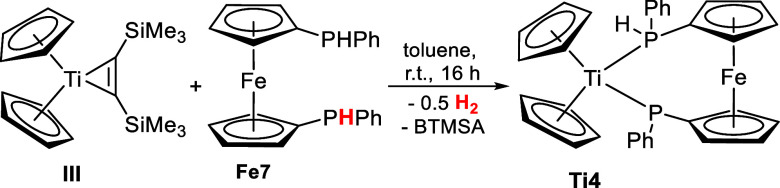
Reaction of Titanocenebis(trimethylsilyl)acetylene Complex **III** with 1,1′-Bis(phenylphosphine)ferrocene **Fe7** to the Phosphidophosphine Complex **Ti4**

We also studied the reactivity of **Fe7** toward the titanocenebis(trimethylsilyl)acetylene complex **III**, which resulted in the phosphidophosphine titanium(III) complex **Ti4** (Scheme 5). This redox reaction occurred via the masked titanocene(II) species by release of BTMSA and the reduction of one proton to hydrogen.

**Figure 7:**
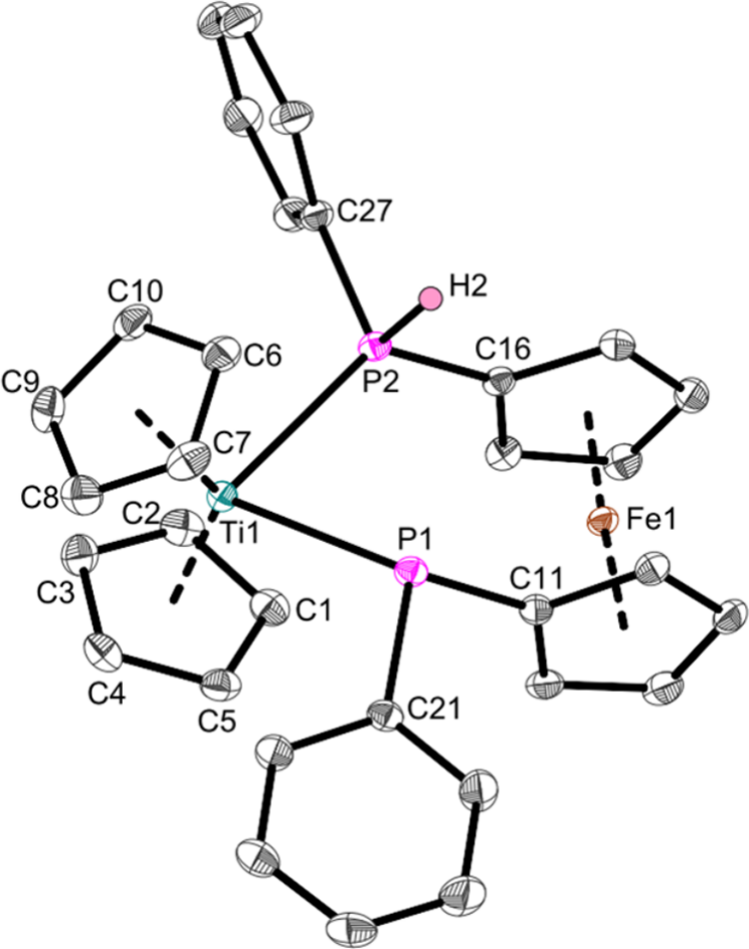
Molecular structure of complex **Ti4**. Displacement ellipsoids are drawn at the 50% probability level. Redundant H atoms have been omitted for clarity. Selected bond lengths (Å) and angles (deg): Ti1–P1 2.6210(5), Ti1–P2 2.6017(5), P1–C11 1.8429(17), P1–C21 1.8419(17), P2–C16 1.7992(16), P1–C27 1.8230(16), P1–Ti1–P2 78.776(15), C11–P1–C21 98.46(8), C16–P2–C27 103.71(7), Ct1–Ti–Ct2 133.9 (Ct1 = Centroid of C1–C5; Ct2 = Centroid of C6–C10).

The structure of the resulting paramagnetic phosphidophosphine complex **Ti4** was revealed via single-crystal X-ray diffraction (Figure 7), showing that only one P–H group was deprotonated.

The Ti–P bond lengths of the phosphido ligand (2.6210(5) Å) and the phosphine ligand (2.6017(5) Å) are rather similar, a feature shared with other phosphidophosphine complexes (2.6040(5), 2.6247(6) Å).^58^ According to the sum of covalent radii (∑r_cov_(Ti–P) = 2.47 Å)^47^ these are elongated single bonds. The asymmetry of the ligand system is evident in the difference of the P–C_Cp_ bonds as the P2–C16 bond of the phosphine ligand is much shorter (1.7992(16) Å) than the P1–C11 bond of the phosphido ligand (1.8429(17) Å) and differs considerably from regular Aryl–P bonds (1.836 Å).^57^ It shows increased double bond character according to the sum of covalent radii (∑r_cov_(P–C) = 1.86 Å,^47^ ∑r_cov_(P=C) = 1.69 Å^48^), a feature also found within **Fe7** and similar ferrocene phosphines (1.804(3), 1.816(3), 1.802(3) Å).^26^

**Figure 8:**
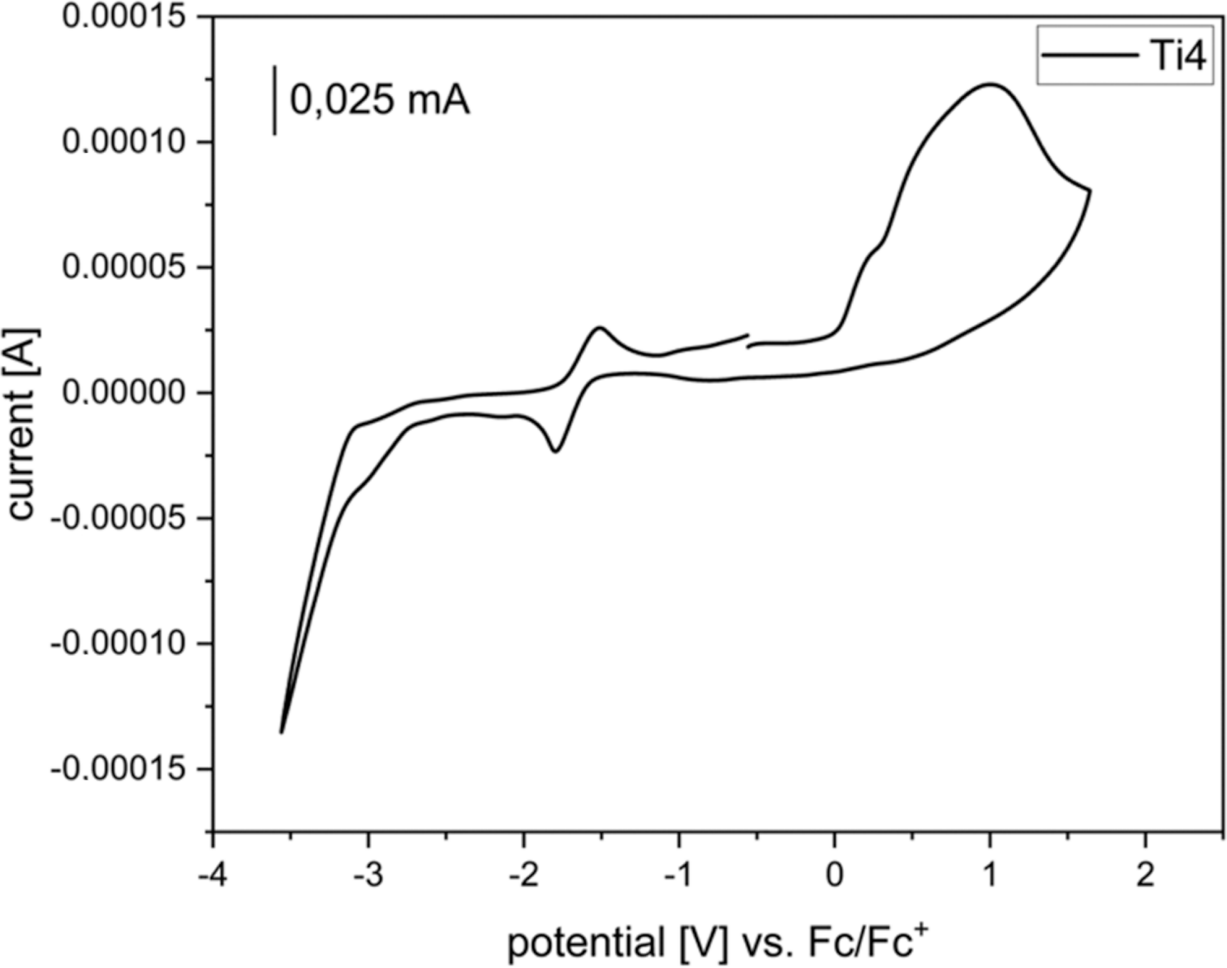
Cyclic voltammogram of complex **Ti4**. Measurements were performed in THF at room temperature under an argon atmosphere (supporting electrolyte [*n*-Bu_4_N]PF_6_, 0.1 M, scan rate 0.1 V/s). Potentials were referenced to the ferrocene/ferrocene^+^ redox couple with *E*_1/2_ = 0.00 V.

Like for **Ti3b**, cyclic voltammetric measurements were performed on **Ti4** (Figure 8). The irreversible oxidation of the ferrocene moiety (Fe(II)/Fe(III) *E*_ox,Fc_ = 0.23 V) is covered by the irreversible oxidation of the phosphine and phosphido ligands (*E*_ox,P_ = 0.99 V), whereas the oxidation of the titanocene moiety (Ti(III)/Ti(IV) *E*_1/2ox,Ti_ = −1.66 V) is reversible. In addition, the irreversible Ti(III)/Ti(II) reduction (*E*_red,Ti_ = −3.03 V) is visible. The multistep oxidation of the ferrocene and the phosphine ligands was also observed for **Fe7** (SI, Figure S23).

**Figure 9:**
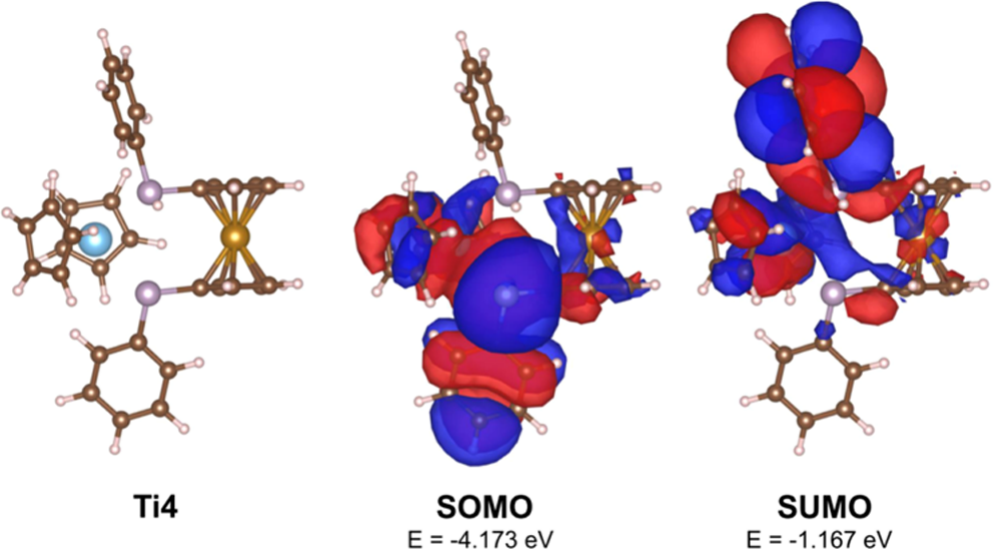
Calculated structure of **Ti4** without (left) and with the calculated surface diagram of the SOMO (middle) and the SUMO (right)(isodensity value 0.01 at B3LYP/Def2-TZVP).

The diffuse SOMO (Figure 9, middle) of **Ti4** is mainly located at the titanocene moiety and the phosphido ligand and partly in the ferrocene moiety. The SUMO (Figure 9, right) is located at the phosphine ligand and partly at both metallocenes.

**Figure 10:**
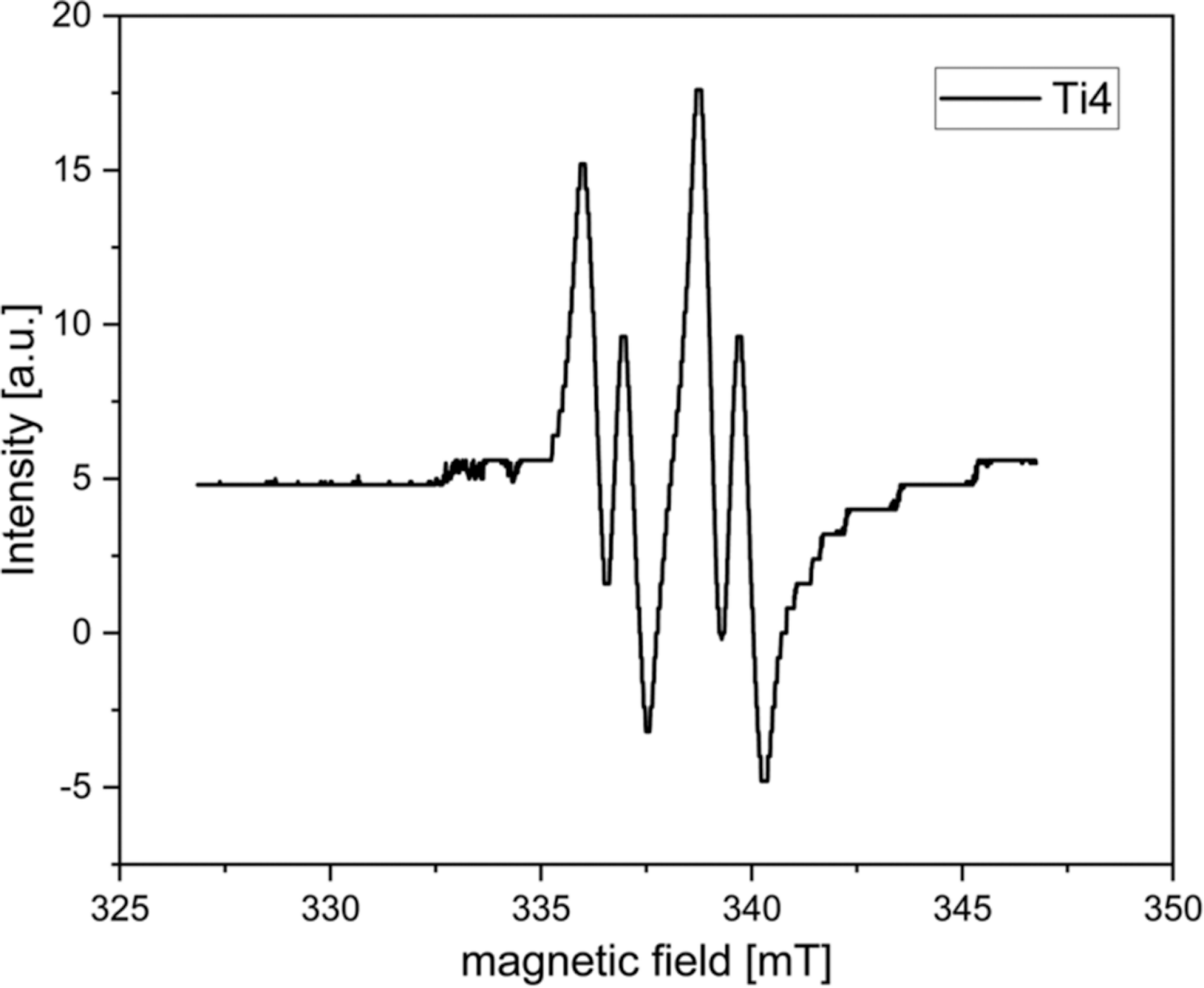
EPR spectrum of complex **Ti4** at room temperature. (*g* = 1.984).

This bonding situation is supported by the EPR spectrum of **Ti4**, showing a double doublet coupling pattern, which correlates with the two different phosphine ligands (Figure 10). A similar coupling pattern was reported for another phosphidophosphine titanium(III) complex.^58^

**Scheme 6:**
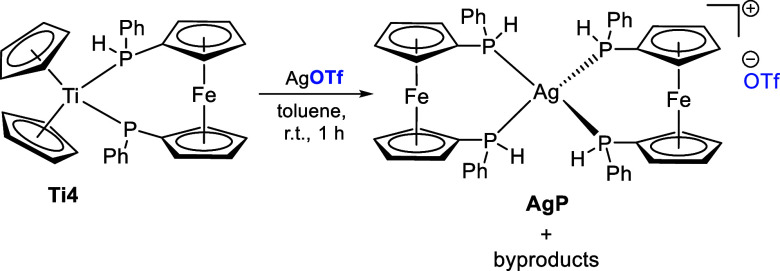
Non-stoichiometric Reaction of **Ti4** with AgOTf to the Silver(I)ferrocenephosphine Triflate Complex **AgP**

We attempted to chemically oxidize **Ti4** with AgOTf. However, this resulted in the formation of the di(1,1′-bis(phenylphosphine)ferrocene) silver(I) triflate complex **AgP** and other byproducts in a nonstoichiometric reaction (Scheme 6).

**Figure 11:**
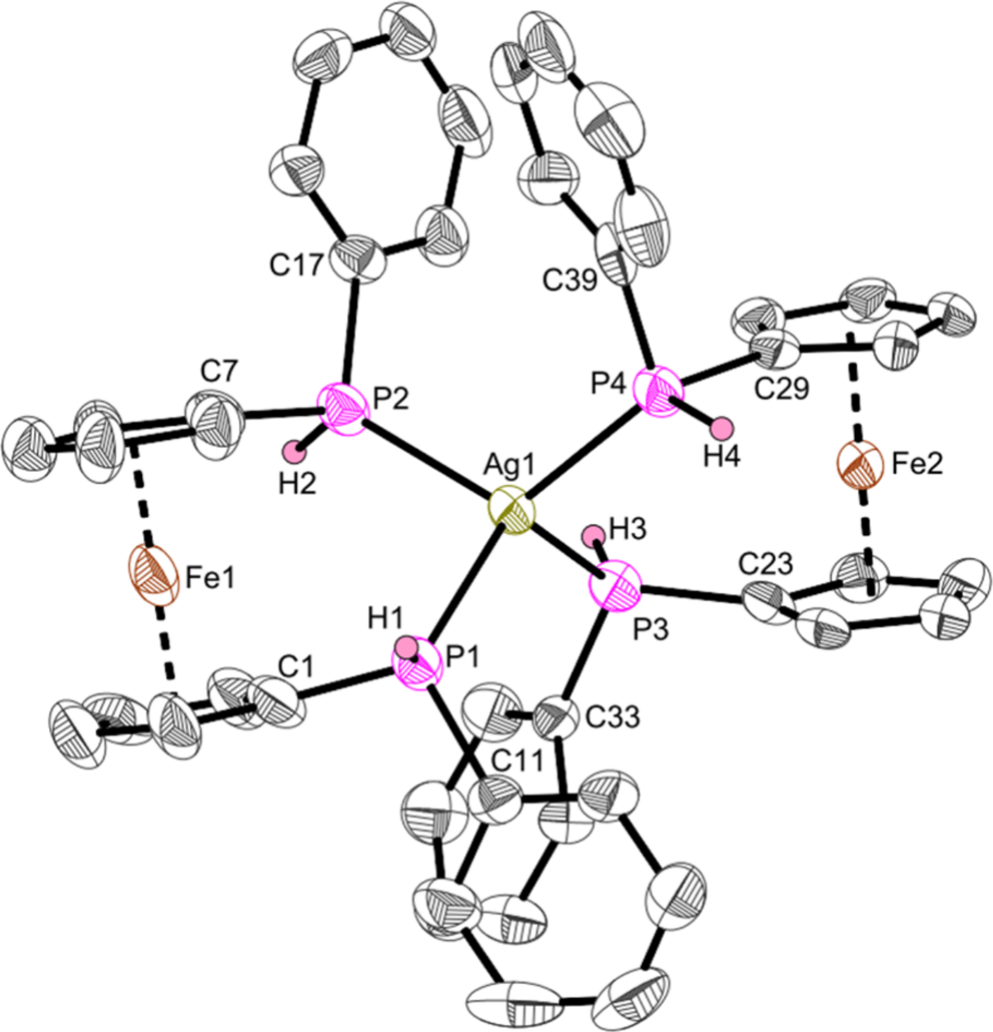
Molecular structure of complex **AgP**. Displacement ellipsoids are drawn at the 50% probability level. Redundant H atoms (apart from H1–4), solvent molecules and the triflate anion have been omitted for clarity. Selected bond lengths (Å) and angles (deg): Ag1–P1 2.476(3), Ag2–P2 2.489(4), Ag1–P3 2.484(4), Ag1–P4 2.476(4), P1–C1 1.780(14), P2–C7 1.784(14), P3–C23 1.791(14), P4–C29 1.812(13), P1–Ag1–P2 102.15(11), P3–Ag1–P4 103.87(13), P1–Ag1–P3 112.99(12), P2–Ag1–P4 118.11(12), P1–Ag1–P4 116.56(12), P2–Ag1–P3 102.62(13).

This was revealed by single-crystal X-ray diffraction as crystals of **AgP** could be obtained from the mixture of products (Figure 11).

The four different Ag–P bonds (2.476(4)–2.489(4) Å) are in the range of a slightly shortened Ag–P single bond according to the sum of covalent radii (∑r_cov_(Ag–P) = 2.52 Å)^59^ and in comparison with other di(bis(phosphine)) silver(I) complexes (2.5604(9),^60^ 2.5385(7),^61^ 2.5498(12) Å^62^). The C_Cp_–P bonds (1.780(14)–1.812(13) Å) are shortened C–P single bonds similar to **Ti4**, **Fe7** and similar ferrocene phosphines (1.804(3), 1.816(3), 1.802(3) Å).^26^ We abstained from a more detailed discussion of bond length, etc. for **AgP** due to poor data quality of the structure.

## Summary and Conclusions

In this work, we showed the syntheses of further tunable ferrocene-containing titanium complexes via insertion of ferrocene aldehydes and ketones or deprotonation of ferrocene alcohols by bis(π–η^5^:σ–η^1^-pentafulvene)titanium complexes. The second pentafulvene moiety was used to introduce further functionalities and generate bimetallic complexes with O- and P-based bridges. Using the bifunctional ferrocene derivatives, double deprotonation occurred to either generate dialcoholate titanium complexes or a P–P-linked ferrocenephosphine. This unprecedented phosphidophosphine titanium complex is the first example of a ferrocene-containing phosphido titanium complex and was analyzed using NMR measurements, single-crystal X-ray diffraction, EPR spectroscopy, cyclic voltammetry and computational data.

## Experimental Section

All reactions were carried out under a dry nitrogen or argon atmosphere using standard Schlenk and glovebox techniques. Caution! Extreme care should be taken both in the handling of the cryogen liquid nitrogen and its use in the Schlenk line trap to avoid the condensation of oxygen from air. Solvents were dried according to standard procedures over Na/K alloy with benzophenone as indicator and subsequently distilled and stored under a nitrogen atmosphere. Bis(π–η^5^:σ–η^1^-pentafulvene)titanium complexes **I** and **II**(52) and titanocenebis(trimethylsilyl)acetylene complex **III**(63) were prepared according to published procedures. Ferrocene derivatives **Fe1**,^45^**Fe2**,^46^**Fe3**,^46^**Fe4**,^64^**Fe5**,^65^**Fe6**,^66^ and **Fe7**(26) were prepared according to published procedures. NMR spectra were recorded on a Bruker AVANCE III 500 spectrometer (^1^H 500 MHz). IR spectra were recorded on a Bruker Tensor 27 spectrometer using an attenuated total reflection (ATR) method. Elemental analyses were carried out on a Euro EA 3000 Elemental Analyzer. Melting points were determined using a Mettler Toledo MP30. EPR spectra were recorded on a Magnettech ESR spectrometer MiniScope MS300. The cyclovoltammetric measurements were performed using Metrohm Autolab PGSTAT204. Platinum was used as the counter electrode, Ag/Ag^+^ as reference electrode and glassy carbon as working electrode. All electrode potentials are referenced versus the internal standard ferrocene/ferrocenium (Fc/Fc^+^) couple. Cyclic voltammetry measurements were performed in a dry and degassed 0.1 M N(*n*-Bu)_4_PF_6_/THF solution at room temperature under an N_2_ atmosphere. All redox potentials are referenced to Fc/Fc^+^. All DFT (density functional theory) calculations were performed with the B3LYP/Def2-TZVP level of theory.

Further exact details of syntheses, crystallographic data, NMR spectra and cyclic voltammograms are given in the Supporting Information (SI).

## Supporting Information Available

NMRspectra, cyclic voltammograms, and crystallographic details (PDF)
Crystal Structures (ZIP)
