# Supplementary material for: Syntheses, Characterization, and Redox Activity of Ferrocene-Containing Titanium Complexes
Source: ACS Omega. 2024 Jun 24;9(26):29017–24. doi: 10.1021/acsomega.4c04332 (PMC11223194; doi:10.1021/acsomega.4c04332)
Supplement: Supplementary file 1 — ao4c04332_si_001.pdf [file ao4c04332_si_001.pdf]

Supporting Information

Syntheses, Characterization and Redox Activity of Ferrocene-Containing Titanium complexes

Kevin Schwitalla, Justin Klimek, Tobias Greven, Marc Schmidtmann and Rüdiger Beckhaus*

Chemistry Department, Carl von Ossietzky University of Oldenburg, 26111 Oldenburg, Germany

E-mail: ruediger.beckhaus@uol.de

TABLE OF CONTENTS

Synthesis and characterization of compounds.. 2

NMR spectra of complexes.. 10

Crystallographic data.. 21

Cyclic voltammograms of precursors.. 24

References 25

Synthesis and characterization of compounds

Synthesis of Ti1a:

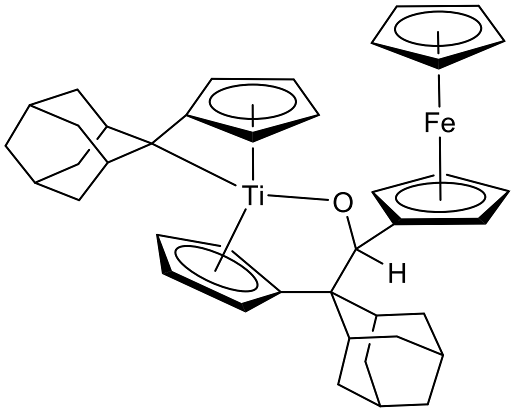

Bis(adamantylidenefulvene)titanium complex **I** (200 mg, 0.450 mmol) and ferrocenealdehyde **Fe1** (96.3 mg, 0.450 mmol) were dissolved in 10 ml of dry *n*-hexane. The reaction mixture was stirred for 16 h at room temperature to give a brown suspension. The solid was isolated from the supernatant and the residue was dried under vacuum to yield the product as a beige solid (mixture of diastereomers 1 : 0.4). Yellow crystals suitable for single-crystal X-ray diffraction analysis precipitated from a slowly evaporating solution of **Ti1a** in C_6D_6 after several days.

Yield: 200 mg, 0.304 mmol, 68%.

^{1}H NMR (C_6D_6, 500 MHz, 305 K): δ = 1.40-2.78 (m, 28 H, Ad-H), 3.87-3.89 (m, 1 H, Fc-H), 3.93-3.95 (m, 1 H, Fc-H), 4.03-4.05 (m, 1 H, Cp-H), 4.09 (s, 5 H, Fc-H), 4.10 (s, 1 H, Fc-H), 4.16-4.18 (m, 1 H, Fc-H), 4.20-4.22 (m, 1 H, Cp-H), 4.60-4.63 (m, 1 H, Cp-H), 5.53-5.57 (m, 1 H, Cp-H), 5.60-5.63 (m, 1 H, Cp-H), 5.76 (s, 1 H, O-CH), 6.09-6.12 (m, 1 H, Cp-H), 6.44-6.47 (m, 1 H, Cp-H), 7.36-7.39 (m, 1 H, Cp-H) ppm.

^{13}C{^{1}H} NMR (C_6D_6, 125 MHz, 305 K): δ = 27.6 (Ad-CH), 28.5 (Ad-CH), 28.8 (Ad-CH), 29.9 (Ad-CH), 32.0 (Ad-CH_2), 33.8 (Ad-CH_2), 34.0 (Ad-CH_2), 34.3 (Ad-CH), 36.1 (Ad-CH), 36.2 (Ad-CH_2), 36.5 (Ad-CH_2), 36.9 (Ad-CH), 37.0 (Ad-CH), 38.7 (Ad-CH_2), 38.8 (Ad-CH_2), 39.3 (Ad-CH_2), 44.1 (Ad-CH_2), 45.9 (Ad-CH_2), 54.8 (O-C-C_{exo}-C_q), 65.5 (Fc-CH), 67.4 (Fc-CH), 69.2 (Fc-CH), 69.3 (5 x Fc-CH), 70.1 (Fc-CH), 95.3 (Fc-C_q), 102.4 (Cp-CH), 103.3 (Cp-CH), 105.6 (O-CH), 105.8 (Cp-CH), 108.9 (Cp-CH), 114.1 (Cp-CH), 114.3 (Cp-CH), 117.6 (Cp-CH), 117.8 (Fv-C_{exo}-C_q), 126.5 (C_{ipso}-C_q), 147.7 (C_{ipso}-C_q) ppm.

IR (ATR): ṽ = 3094, 2900, 2845, 2662, 2360, 2341, 1472, 1447, 1261, 1104, 1064, 1040, 1019, 1000, 805, 774, 752, 717, 690, 669, 617, 592, 581 cm^{-1}.

Melting point: 160 – 165 °C.

EA: calcd. for $C_{41}H_{46}FeOTi$: C 74.78, H 7.04. Found: C 74.42, H 7.01.

Synthesis of Ti1b:

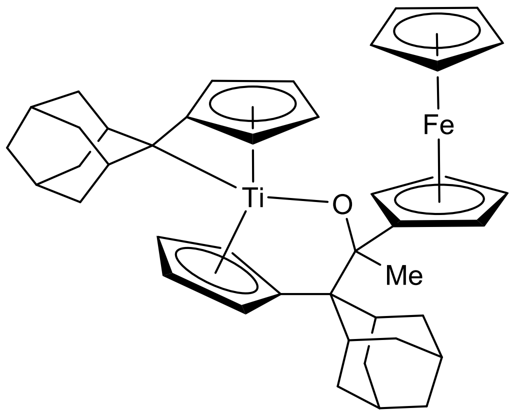

Bis(adamantylidenefulvene)titanium complex **I** (200 mg, 0.450 mmol) and acetylferrocene **Fe2** (102.6 mg, 0.450 mmol) were dissolved in 10 ml of dry *n*-hexane. The reaction mixture was stirred for 16 h at room temperature to give a brown suspension. The solid was isolated from the supernatant and the residue was dried under vacuum to yield the product (mixture of diastereomers 1 : 0.2) as a beige solid.

Yield: 152 mg, 0.226 mmol, 50%.

^{1}H NMR (C_6D_6, 500 MHz, 305 K): δ = 1.45-2.85 (m, 28 H, Ad-H), 1.58 (s, 3 H, CH_3), 3.90-3.92 (m, 1 H, Fc-H), 3.99-4.01 (m, 1 H, Fc-H), 4.04 (s, 1 H, Fc-H), 4.05-4.07 (m, 1 H, Cp-H), 4.08 (s, 5 H, Fc-H), 4.09-4.10 (m, 1 H, Fc-H), 4.38-4.41 (m, 1 H, Cp-H), 4.43-4.45 (m, 1 H, Cp-H), 5.45-5.48 (m, 1 H, Cp-H), 5.58-5.61 (m, 1 H, Cp-H), 6.06-6.09 (m, 1 H, Cp-H), 6.20-6.22 (m, 1 H, Cp-H), 7.17-7.19 (m, 1 H, Cp-H) ppm.

^{13}C{^{1}H} NMR (C_6D_6, 125 MHz, 305 K): δ = 27.8 (Ad-CH), 28.2 (Ad-CH), 28.8 (Ad-CH), 29.9 (Ad-CH), 31.8 (Ad-CH), 32.0 (Ad-CH), 33.0 (Ad-CH_2), 33.3 (Ad-CH_2), 33.4 (Ad-CH), 35.2 (Ad-CH_2), 35.9 (Ad-CH), 36.3 (Ad-CH_2), 37.5 (Ad-CH_2), 38.0 (Ad-CH_2), 38.7 (Ad-CH_2), 38.9 (CH_3), 39.6 (Ad-CH_2), 43.5 (Ad-CH_2), 45.9 (Ad-CH_2), 56.6 (O-C-C_{exo}-C_q), 66.4 (Fc-CH), 67.8 (Fc-CH), 68.3 (Fc-CH), 68.9 (Fc-CH), 69.2 (5 x Fc-CH), 101.2 (Cp-CH), 102.2 (Fc-C_q), 102.8 (O-C-C_q), 104.1 (Cp-CH), 104.9 (Cp-CH), 107.2 (Cp-CH), 108.8 (Cp-CH), 113.7 (Cp-CH), 114.0 (Cp-CH), 118.0 (Fv-C_{exo}-C_q), 118.5 (Cp-CH), 127.0 (C_{ipso}-C_q), 147.6 (C_{ipso}-C_q) ppm.

IR (ATR): $\tilde{\nu}$ = 2907, 2848, 2360, 2339, 1456, 1112, 1081, 1055, 1025, 944, 805, 759, 617 cm^{-1}.

Melting point: 175 – 180 °C.

EA: calcd. for $C_{42}H_{48}FeOTi$: C 75.01, H 7.19. Found: C 75.26, H 7.66.

Synthesis of Ti1c:

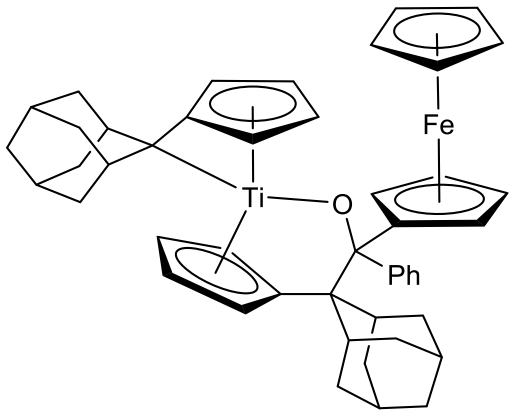

Bis(adamantylidenefulvene)titanium complex **I** (200 mg, 0.450 mmol) and benzoylferrocene **Fe3** (130.6 mg, 0.450 mmol) were dissolved in 10 ml of dry *n*-hexane. The reaction mixture was stirred for 16 h at room temperature to give a green suspension. The solid was isolated from the supernatant and the residue was dried under vacuum to yield the product (mixture of diastereomers 1 : 0.25) as a green solid.

Yield: 299 mg, 0.407 mmol, 90%.

^{1}H NMR (C_6D_6, 500 MHz, 305 K): δ = 1.35-2.90 (m, 28 H, Ad-H), 3.79 (s, 5 H, Fc-H), 4.07-4.13 (m, 3 H, 2 x Fc-H, Cp-H), 4.21-4.23 (m, 1 H, Cp-H), 4.35-4.38 (m, 1 H, Fc-H), 4.54-4.57 (m, 1 H, Fc-H), 4.60-4.63 (m, 1 H, Cp-H), 5.44-5.48 (m, 1 H, Cp-H), 5.55-5.58 (m, 1 H, Cp-H), 6.15-6.18 (m, 1 H, Cp-H), 6.55-6.58 (m, 1 H, Cp-H), 7.18-7.24 (m, 3 H, Ph-H), 7.41-7.44 (m, 1 H, Cp-H), 7.71-7.79 (m, 2 H, Ph-H) ppm.

^{13}C{^{1}H} NMR (C_6D_6, 125 MHz, 305 K): δ = 27.3 (Ad-CH), 27.9 (Ad-CH), 28.7 (Ad-CH), 30.0 (Ad-CH), 32.0 (Ad-CH_2), 32.6 (Ad-CH_2), 34.2 (Ad-CH_2), 34.7 (Ad-CH), 35.7 (Ad-CH), 35.8 (Ad-CH), 36.4 (Ad-CH), 37.3 (Ad-CH_2), 38.4 (Ad-CH_2), 38.5 (Ad-CH_2), 38.9 (Ad-CH_2), 39.2 (Ad-CH_2), 44.8 (Ad-CH_2), 45.9 (Ad-CH_2), 61.0 (O-C-C_{exo}-C_q), 64.8 (Fc-CH), 69.2 (Fc-CH), 69.6 (5 x Fc-CH), 71.4 (Fc-CH), 73.6 (Fc-CH), 95.5 (Fc-C_q), 102.4 (Cp-CH), 105.0 (Cp-CH), 105.3 (Cp-CH), 106.2 (Cp-CH), 107.5 (Cp-CH), 111.9 (Cp-CH), 113.1 (O-C-C_q), 116.7 (Cp-CH), 117.1 (Fv-C_{exo}-C_q), 118.8 (Cp-CH), 126.2 (Ph-CH), 126.4 (Ph-CH), 126.6 (C_{ipso}-C_q), 128.4 (Ph-CH), 128.5 (Ph-CH), 129.5 (Ph-CH), 148.3 (C_{ipso}-C_q), 149.7 (Ph-C_q) ppm.

IR (ATR): ṽ = 3094, 2900, 2845, 2663, 2362, 2341, 1639, 1462, 1445, 1261, 1096, 1071, 1044, 1033, 1018, 797, 774, 757, 741, 717, 692, 650, 583 cm^{-1}.

Melting point: 95 – 100 °C.

EA: calcd. for $C_{41}H_{46}FeOTi$: C 76.84, H 6.86. Found: C 77.58, H 6.48.

Synthesis of Ti2a:

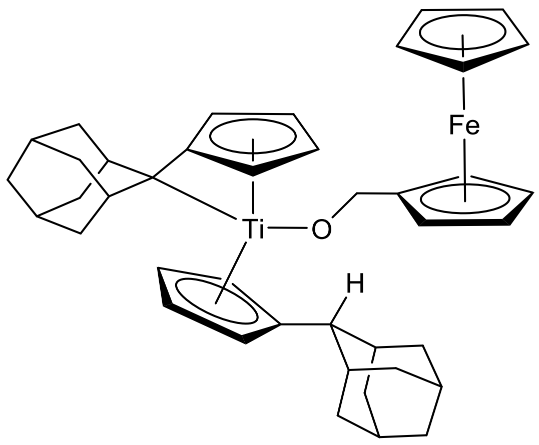

Bis(adamantylidenefulvene)titanium complex **I** (150 mg, 0.337 mmol) and ferrocenemethanol **Fe4** (72.9 mg, 0.337 mmol) were dissolved in 10 ml of dry *n*-hexane. The reaction mixture was stirred for 16 h at room temperature to give a brown suspension. The solid was isolated from the supernatant and the residue was dried under vacuum to yield the product as a beige solid. Yellow crystals suitable for single-crystal X-ray diffraction analysis precipitated from a slowly evaporating solution of **Ti2a** in C_6D_6 after several days.

Yield: 133 mg, 0.201 mmol, 60%.

^{1}H NMR (C_6D_6, 500 MHz, 305 K): δ = 1.42-2.46 (m, 28 H, Ad), 2.99 (s, 1 H, C_{exo}H), 3.93-3.95 (m, 2 H, Fc-H), 3.96-3.97 (m, 5 H, Fc-H), 4.00-4.03 (m, 2 H, Fc-H), 4.06-4.07 (m, 1 H, Cp-H), 4.62-4.67 (m, 2 H, O-CH_2), 4.95-4.98 (m, 1 H, Cp-H), 5.24-5.27 (m, 1 H, Cp-H), 5.35-5.38 (m, 1 H, Cp-H), 5.48-5.51 (m, 1 H, Cp-H), 5.69-5.72 (m, 1 H, Cp-H), 6.02-6.04 (m, 1 H, Cp-H), 6.47-6.49 (m, 1 H, Cp-H) ppm.

^{13}C{^{1}H} NMR (C_6D_6, 125 MHz, 305 K): δ = 28.6 (Ad-CH), 28.6 (Ad-CH), 28.8 (Ad-CH), 29.9 (Ad-CH), 32.4 (Ad-CH), 33.0 (Ad-CH), 33.0 (Ad-CH_2), 33.1 (Ad-CH_2), 36.0 (Ad-CH), 36.1 (Ad-CH), 36.9 (Ad-CH_2), 38.4 (Ad-CH_2), 38.5 (Ad-CH_2), 38.7 (Ad-CH_2), 39.3 (Ad-CH_2), 39.3 (Ad-CH_2), 44.1 (Ad-CH_2), 44.2 (C_{exo}H), 45.6 (Ad-CH_2), 68.0 (Fc-CH), 68.1 (Fc-CH), 68.7 (5 x Fc-CH), 69.0 (Fc-CH), 69.0 (Fc-CH), 73.2 (Fc-CH), 90.3 (Fc-C_q), 104.4 (Cp-CH), 105.9 (Cp-CH), 106.1 (Cp-CH), 108.3 (Cp-CH), 111.7 (Cp-CH), 113.4 (Cp-CH), 113.7 (Cp-CH), 116.0 (Cp-CH), 118.9 (Fv-C_{exo}-C_q), 124.2 (C_{ipso}-C_q), 134.7 (C_{ipso}-C_q) ppm.

IR (ATR): $\tilde{v}$ = 2898, 2847, 2359, 2332, 2159, 2150, 1448, 1116, 1106, 1063, 1032, 802, 775, 761, 691, 669 cm^{-1}.

Melting point: 155 – 160 °C.

EA: calcd. for $C_{41}H_{48}FeOTi$: C 74.55, H 7.32. Found: C 74.28, H 7.08.

Synthesis of Ti2b:

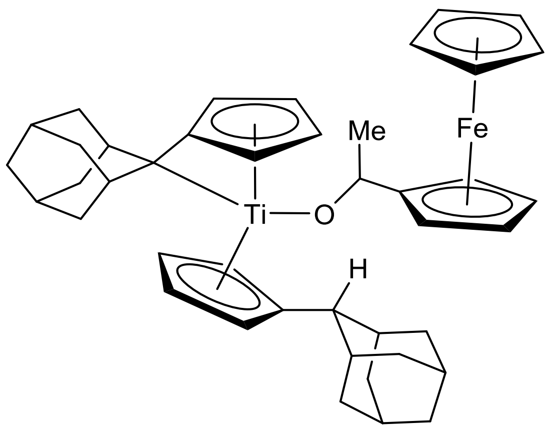

Bis(adamantylidenefulvene)titanium complex **I** (200 mg, 0.450 mmol) and 1-(ferrocenyl)-ethanol **Fe5** (104 mg, 0.450 mmol) were dissolved in 10 ml of dry *n*-hexane. The reaction mixture was stirred for 16 h at room temperature to give a green suspension. The solid was isolated from the supernatant and the residue was dried under vacuum to yield the product (mixture of diastereomers 1 : 0.8) as a green solid. Brown crystals suitable for single-crystal X-ray diffraction analysis precipitated from a slowly evaporating solution of **Ti2b** in C_6D_6 after several days.

Yield: 254 mg, 0.377 mmol, 84%.

^{1}H NMR (C_6D_6, 500 MHz, 305 K): δ = 1.26 (d, 3 H, Me), 1.45-2.48 (m, 28 H, Ad), 2.93 (s, 1 H, C_{exo}H), 3.85-3.88 (m, 1 H, Fc-H), 3.91-3.93 (m, 1 H, Fc-H), 3.94-3.96 (m, 1 H, Fc-H), 3.98 (s, 5 H, Fc-H), 4.04-4.06 (m, 1 H, Fc-H), 4.06-4.09 (m, 1 H, Cp-H), 4.71-4.77 (m, 1 H, O-<u>CH</u>-Me), 5.04-5.08 (m, 1 H, Cp-H), 5.36-5.40 (m, 1 H, Cp-H), 5.41-5.44 (m, 1 H, Cp-H), 5.59-5.62 (m, 1 H, Cp-H), 6.03-6.06 (m, 1 H, Cp-H), 6.15-6.18 (m, 1 H, Cp-H), 6.39-6.41 (m, 1 H, Cp-H) ppm.

^{13}C{^{1}H} NMR (C_6D_6, 125 MHz, 305 K): δ = 26.2 (CH_3), 28.6 (2 x Ad-CH), 28.9 (Ad-CH), 29.9 (Ad-CH), 32.1 (Ad-CH), 32.6 (Ad-CH), 33.0 (Ad-CH_2), 33.2 (Ad-CH), 36.1 (Ad-CH), 36.3 (Ad-CH_2), 37.0 (Ad-CH_2), 38.5 (Ad-CH_2), 38.5 (Ad-CH_2), 38.7 (Ad-CH_2), 39.0 (Ad-CH_2), 39.1 (Ad-CH_2), 43.9 (C_{exo}H), 44.2 (Ad-CH_2), 45.6 (Ad-CH_2), 65.6 (Fc-CH), 67.3 (Fc-CH), 67.7 (Fc-CH), 68.6 (Fc-CH), 68.9 (5 x Fc-CH), 77.5 (O-CH), 95.5 (Fc-C_q), 104.7 (Cp-CH), 105.9 (Cp-CH), 106.3 (Cp-CH), 107.8 (Cp-CH), 111.6 (Cp-CH), 113.3 (Cp-CH), 113.6 (Cp-CH), 116.0 (Cp-CH), 118.6 (Fv-C_{exo}-C_q), 124.0 (C_{ipso}-C_q), 133.9 (C_{ipso}-C_q) ppm.

IR (ATR): ṽ = 2898, 2845, 2358, 2337, 1448, 1099, 1078, 1059, 798, 779, 758, 721, 576 cm^{-1}.

Melting point: 150 – 155 °C.

EA: calcd. for $C_{42}H_{50}FeOTi$: C 74.78, H 7.47. Found: C 74.05, H 7.54.

Synthesis of Ti3a:

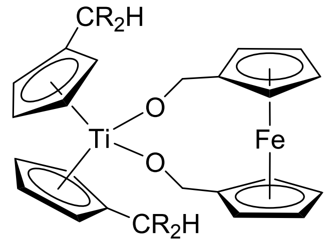

CR_2 = Ad

Bis(adamantylidenefulvene)titanium complex **I** (200 mg, 0.450 mmol) and 1,1'-Ferrocenedimethanol **Fe6** (108.9 mg, 0.450 mmol) were dissolved in 10 ml of dry toluene. The reaction mixture was stirred for 16 h at room temperature to give a brown suspension. The solid was isolated from the supernatant and the residue was dried under vacuum to yield the product as a brown solid.

Yield: 231 mg, 0.335 mmol, 74%.

^{1}H NMR (C_6D_6, 500 MHz, 305 K): δ = 1.56-2.38 (m, 28 H, Ad), 2.90 (s, 2 H, $C_{exo}H$), 4.09-4.15 (m, 8 H, Fc-H), 5.14 (s, 4 H, O-CH_2), 5.94-6.00 (m, 8 H, Cp-H) ppm.

^{13}C{^{1}H} NMR (C_6D_6, 125 MHz, 305 K): δ = 28.4 (4 x Ad-CH), 28.5 (4 x Ad-CH), 32.4 (4 x Ad-CH_2), 33.3 (2 x Ad-CH_2), 39.1 (4 x Ad-CH_2), 44.1 (2 x $C_{exo}H$), 66.5 (4 x Fc-CH), 67.4 (4 x Fc-CH), 75.5 (2 x CH_2), 94.3 (2 x Fc-C_q), 109.2 (4 x Cp-CH), 112.9 (4 x Cp-CH), 140.2 (2 x C_{ipso}-C_q) ppm.

IR (ATR): ṽ = 2899, 2847, 2358, 2341, 1449, 1235, 1098, 1062, 1040, 1020, 985, 922, 802, 740, 627, 576 cm^{-1}.

Melting point: 95 – 100 °C (dec.)

EA: calcd. for $C_{42}H_{50}FeO_2Ti$: C 73.05, H 7.30. Found: C 73.34, H 7.44.

Synthesis of Ti3b:

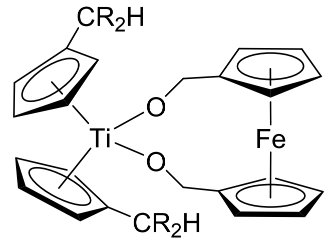

R = *p*-Tol

Bis(di-*para*-tolylfulvene)titanium complex **II** (200 mg, 0.354 mmol) and 1,1'-Ferrocenedimethanol **Fe6** (87.1 mg, 0.354 mmol) were dissolved in 10 ml of dry toluene. The reaction mixture was stirred for 16 h at room temperature to give a brown suspension. The solid was isolated from the supernatant and the residue was dried under vacuum to yield the product as a brown solid.

Yield: 240 mg, 0.296 mmol, 84%.

^{1}H NMR (C_6D_6, 500 MHz, 305 K): δ = 2.14 (s, 12 H, *p*-Tol-CH_3), 4.07-4.13 (m, 8 H, Fc-H), 5.32 (s, 4 H, O-CH_2), 5.43 (s, 2 H, C_{exo}H), 5.60-5.64 (m, 4 H, Cp-H), 5.71-5.73 (m, 4 H, Cp-H), 6.98-7.01 (m, 8 H, Ar-H), 7.13-7.15 (m, 4 H, Ar-H) ppm.

^{13}C{^{1}H} NMR (C_6D_6, 125 MHz, 305 K): δ = 21.0 (4 x *p*-Tol-CH_3), 51.3 (2 x C_{exo}H), 66.9 (4 x Fc-CH), 67.6 (4 x Fc-CH), 76.5 (2 x CH_2), 94.0 (2 x Fc-C_q), 112.2 (8 x Cp-CH), 129.2 (8 x Ar-CH), 129.7 (8 x Ar-CH), 135.8 (4 x Me-Ar-C_q), 138.2 (4 x Ar-C_q), 142.3 (2 x C_{ipso}-C_q) ppm.

IR (ATR): ṽ = 2912, 2859, 1510, 1450, 1234, 1040, 1021, 986, 806, 762, 634, 575 cm^{-1}.

Melting point: 100 – 105 °C.

EA: calcd. for $C_{52}H_{50}FeO_2Ti$: C 77.04, H 6.22. Found: C 77.32, H 6.38.

Synthesis of Ti4:

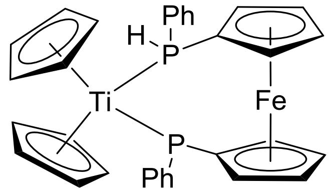

Titanocenbis(trimethylsilyl)acetylene titanium complex **III** (200 mg, 0.574 mmol) and 1,1’-bis(phenylphosphino)ferrocene **Fe7** (231 mg, 0.574 mmol) were dissolved in 10 ml of dry toluene. The reaction mixture was stirred for 16 h at room temperature to give a brown solution. The solvent was removed under reduced pressure and the residue was washed with 10 mL of *n*-hexane. All volatile components were removed under reduced pressure and the residue was dried under vacuum to yield the product as a yellow solid. Yellow crystals suitable for single-crystal X-ray diffraction analysis precipitated from a slowly evaporating solution of **Ti4c** in C_6D_6 after several days.

Yield: 256 mg, 0.442 mmol, 77%.

IR (ATR): $\tilde{\nu}$ = 3066, 2952, 2271, 1688, 1585, 1480, 1433, 1383, 1363, 1311, 1244, 1199, 1166, 1123, 1099, 1066, 1050, 1025, 997, 911, 882, 826, 789, 741, 720, 692, 632, 577 cm^{-1}.

Melting point: 80 °C.

EA: calcd. for $C_{32}H_{29}FeP_2Ti$: C 66.35, H 5.05. Found: C 66.12, H 4.86.

EPR: g = 1.984

NMR Spectra of complexes

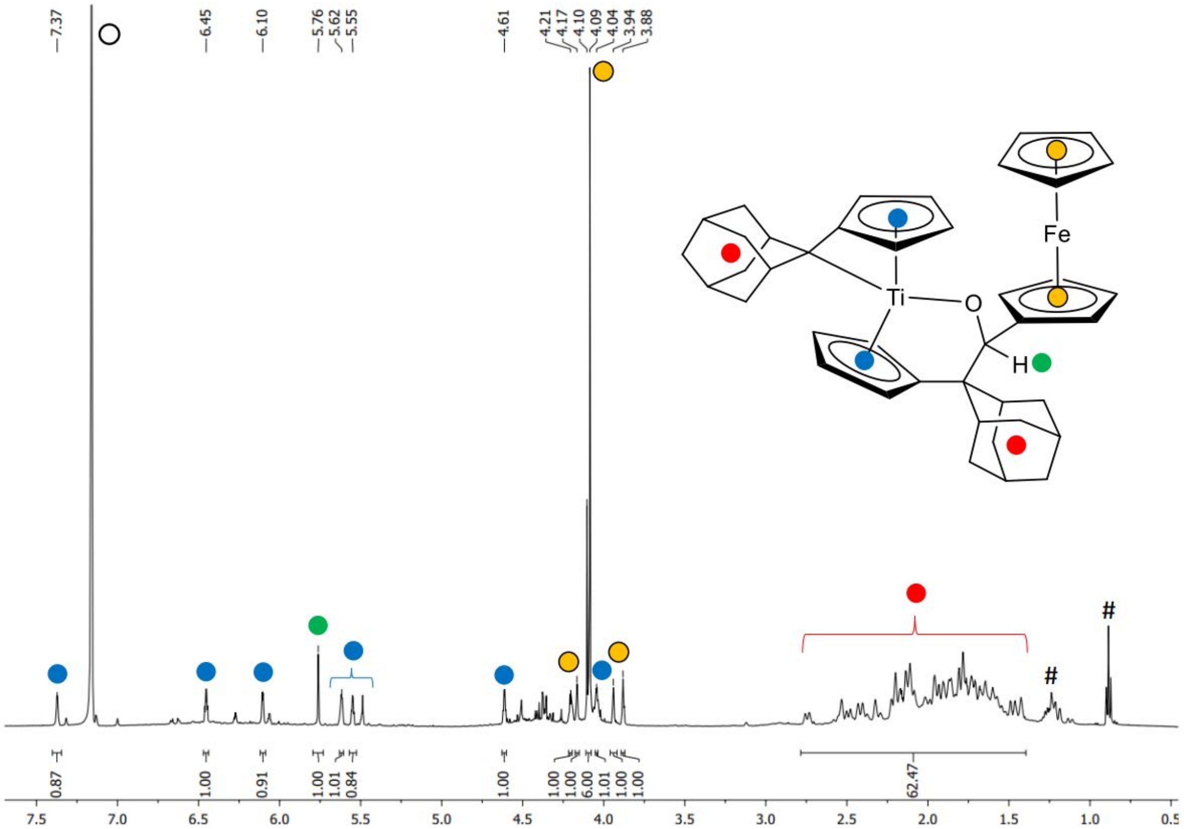

Figure S1: ^{1}H NMR spectrum (500 MHz, C_6D_6, 305 K) of **Ti1a**. Product signals of the main diastereomer are given in colours (° = C_6D_5H, # = *n*-hexane).

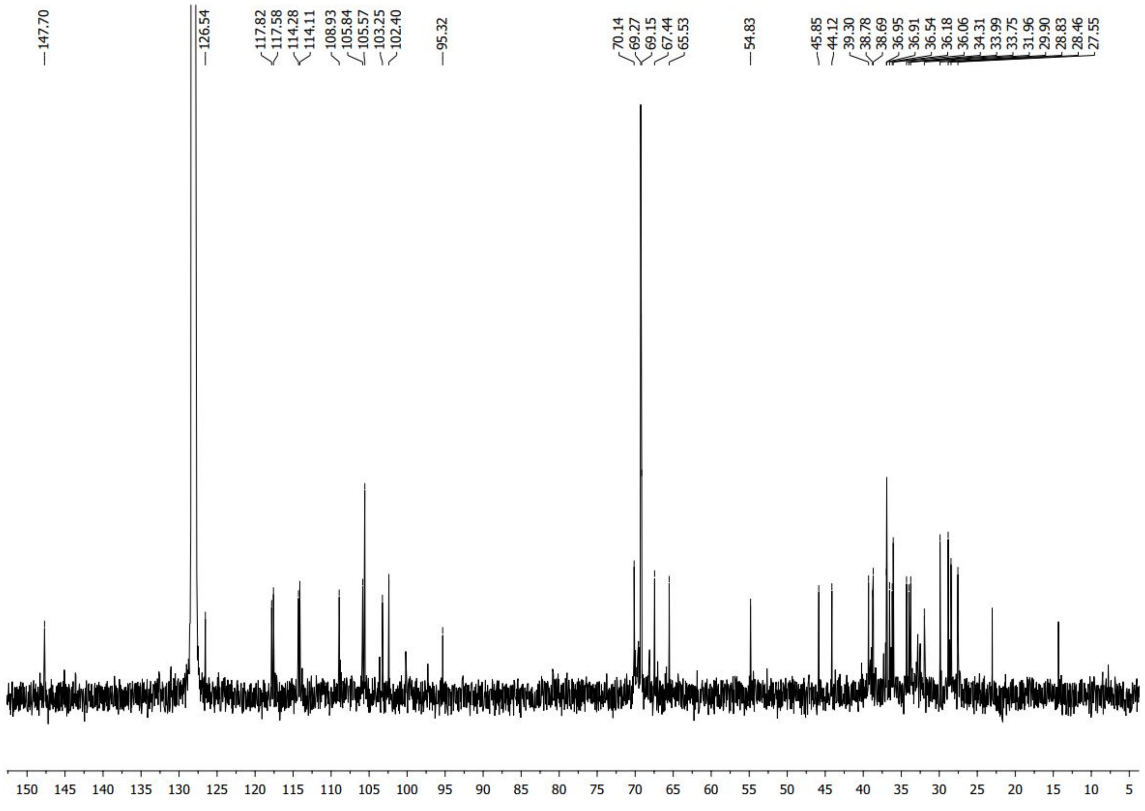

Figure S2: $^{13}C\{^{1}H\}$ NMR spectrum (125 MHz, C_6D_6, 305 K) of **Ti1a**.

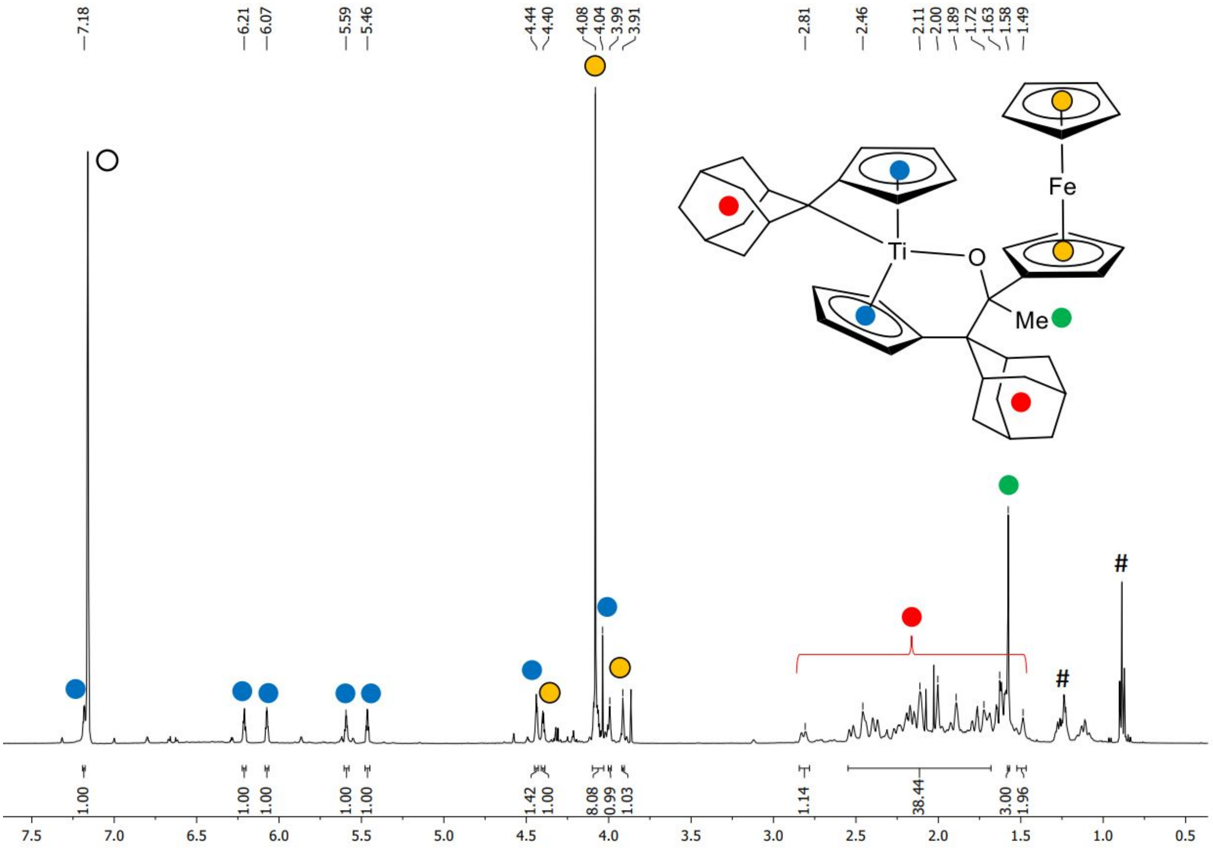

Figure S3: ^{1}H NMR spectrum (500 MHz, C_6D_6, 305 K) of **Ti1b**. Product signals of the main diastereomer are given in colours (^{o} = C_6D_5H, # = *n*-hexane).

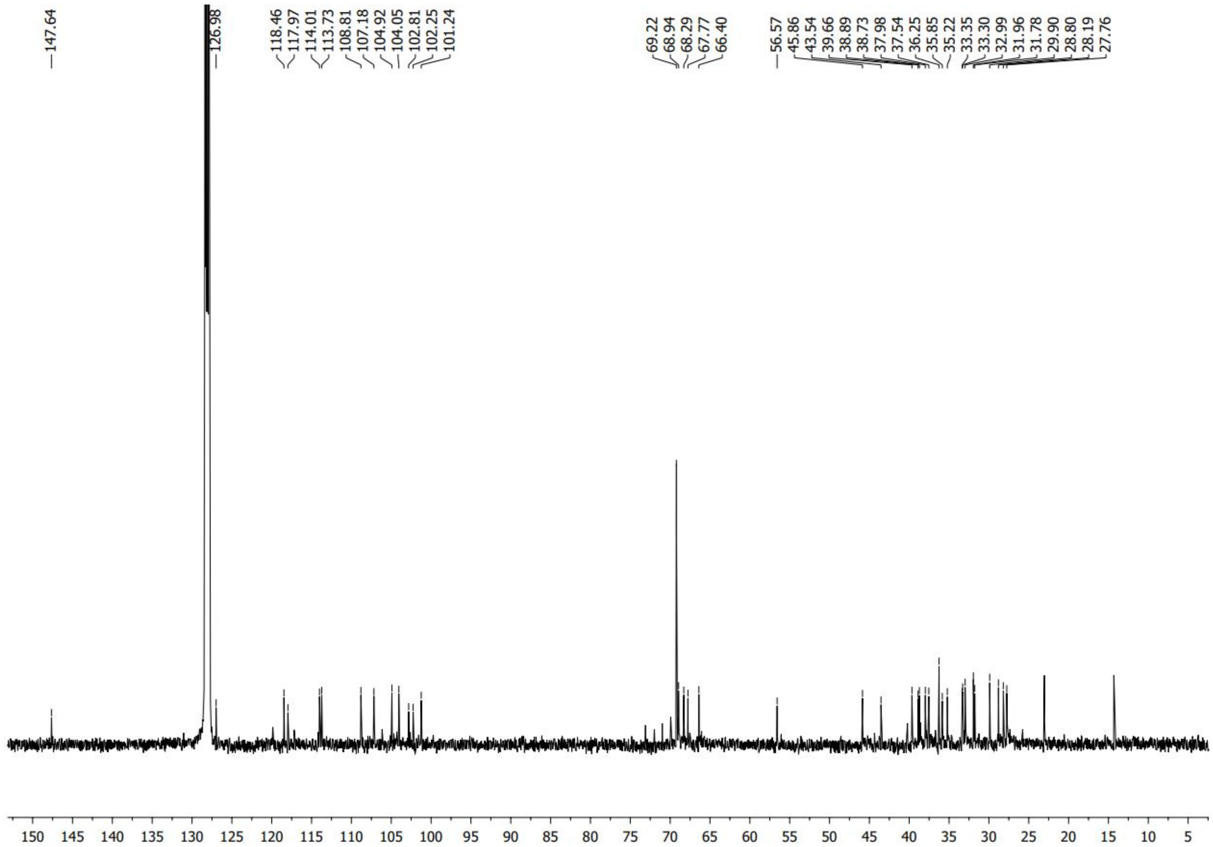

Figure S4: $^{13}C\{^{1}H\}$ NMR spectrum (125 MHz, C_6D_6, 305 K) of **Ti1b**.

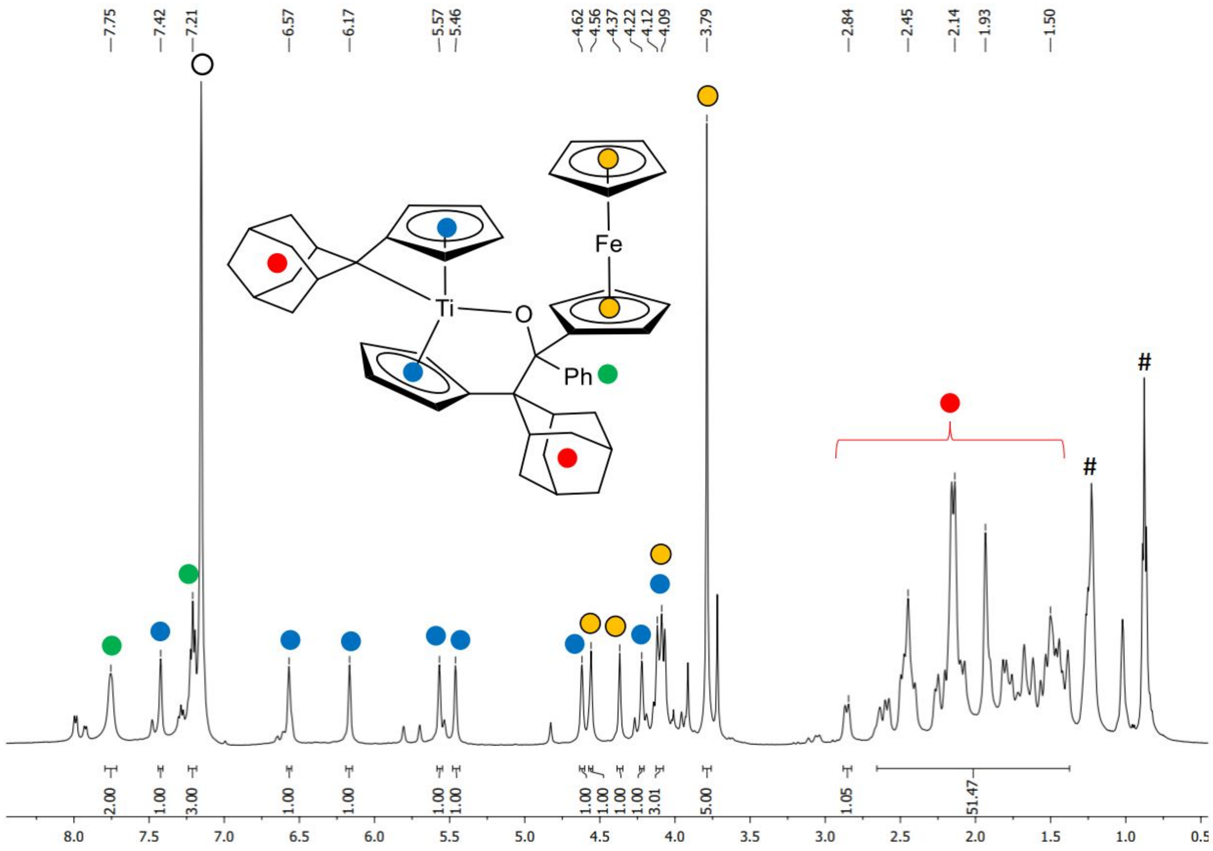

Figure S5: ^{1}H NMR spectrum (500 MHz, C_6D_6, 305 K) of **Ti1c**. Product signals of the main diastereomer are given in colours (^{o} = C_6D_5H, # = *n*-hexane).

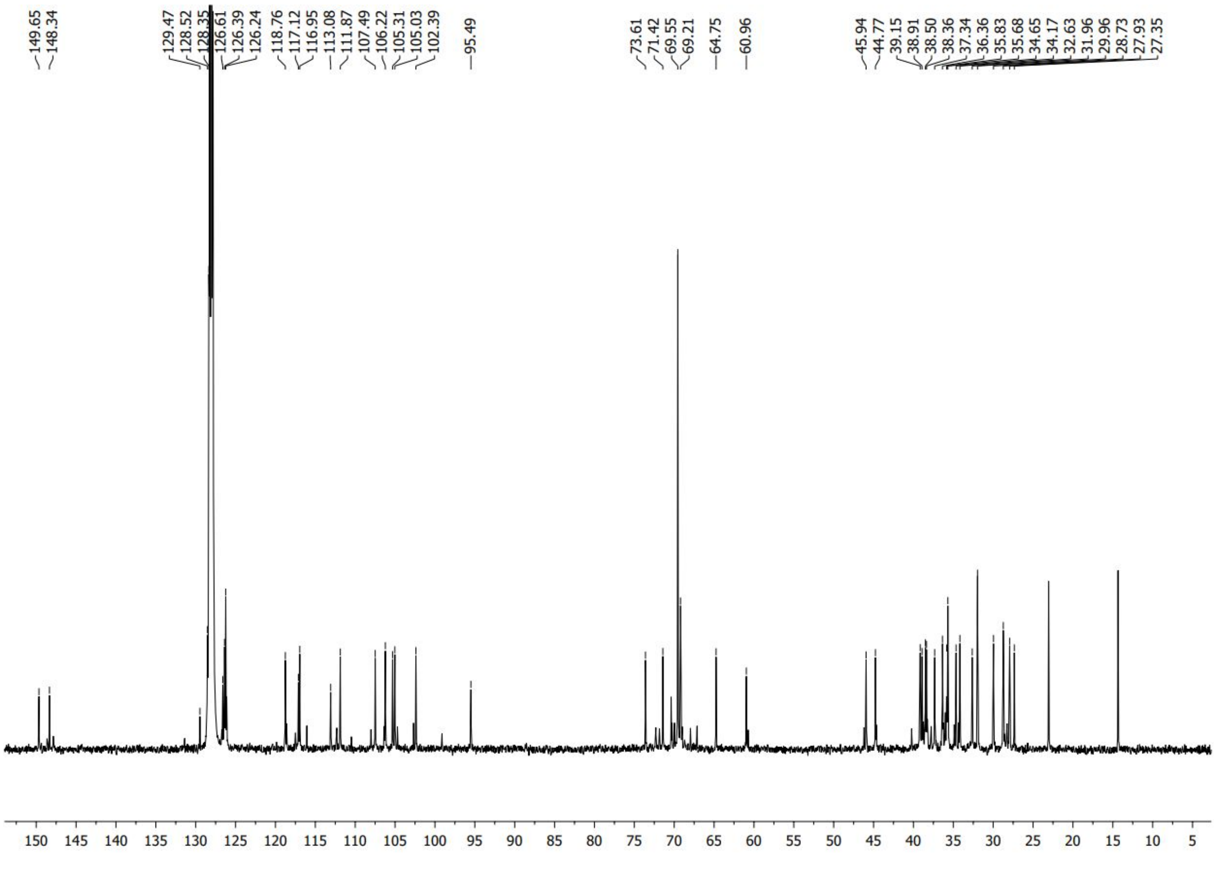

Figure S6: $^{13}C\{^{1}H\}$ NMR spectrum (125 MHz, C_6D_6, 305 K) of **Ti1c**.

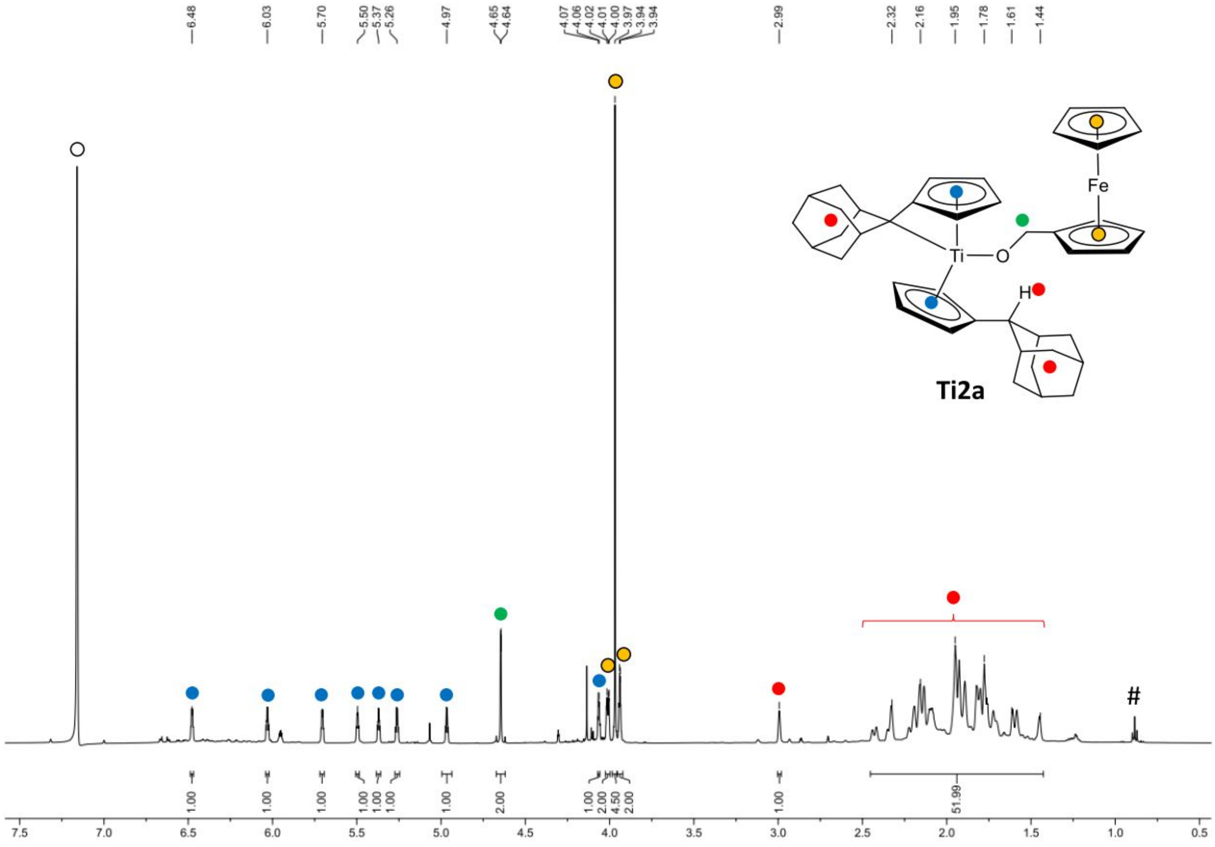

Figure S7: ^{1}H NMR spectrum (500 MHz, C_6D_6, 305 K) of **Ti2a**. Product signals are given in colours (° = C_6D_5H, # = *n*-hexane).

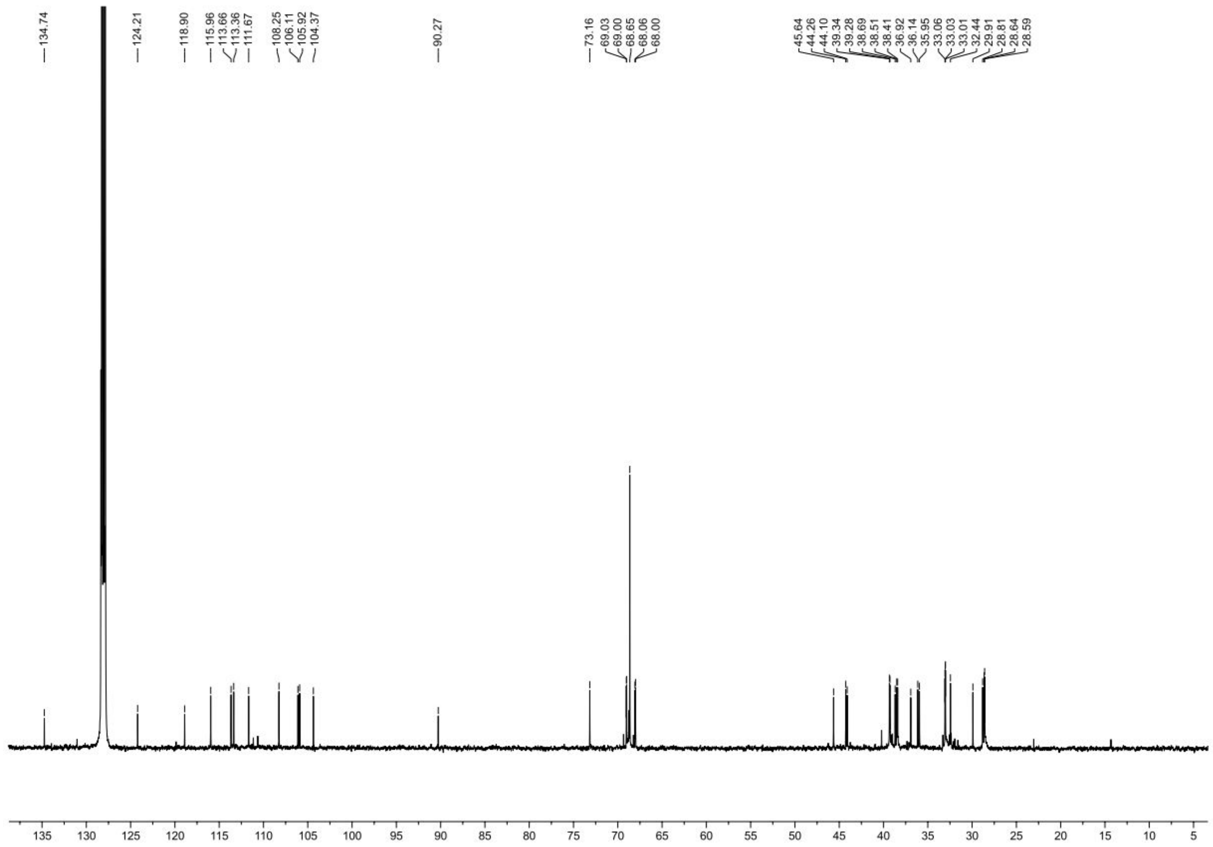

Figure S8: ^{13}C NMR spectrum (125 MHz, C_6D_6, 305 K) of **Ti2a**.

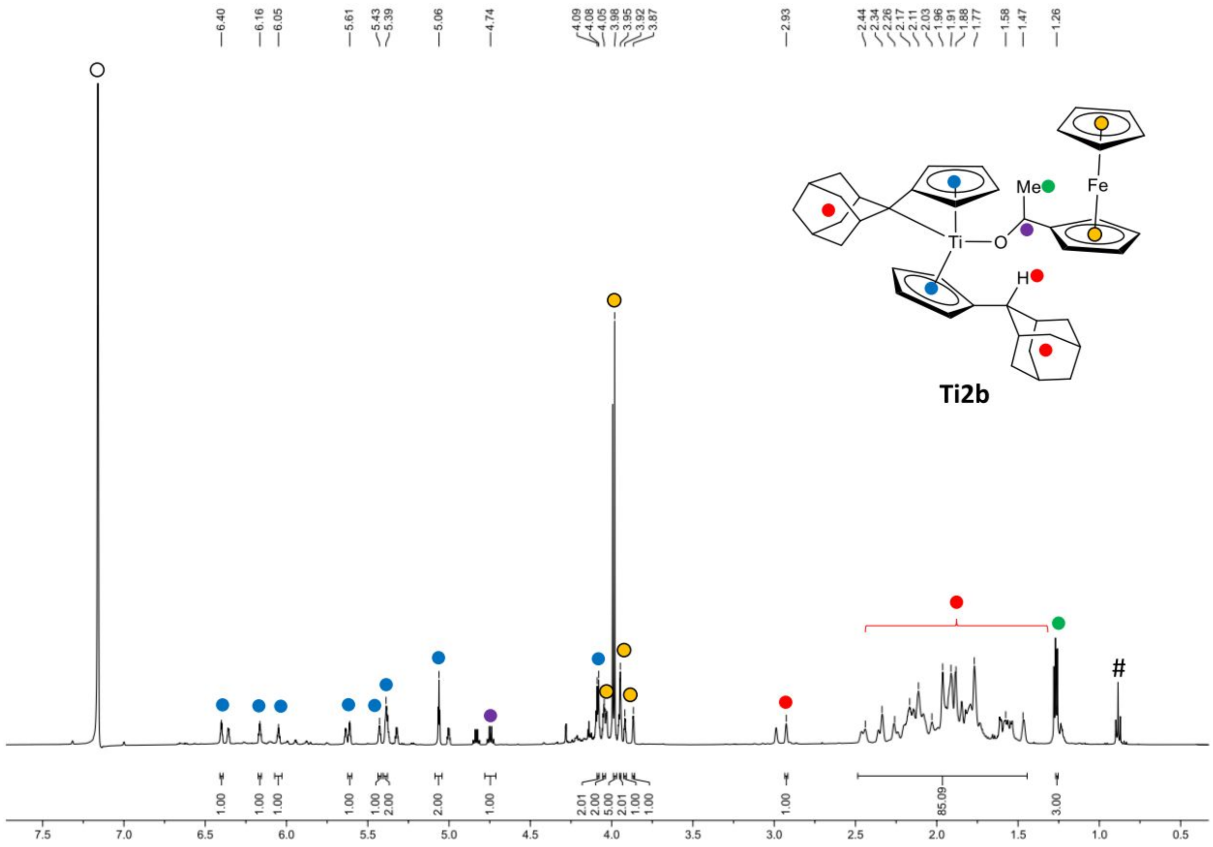

Figure S9: ^{1}H NMR spectrum (500 MHz, C_6D_6, 305 K) of **Ti2b**. Product signals of the main diastereomer are given in colours (° = C_6D_5H, # = *n*-hexane).

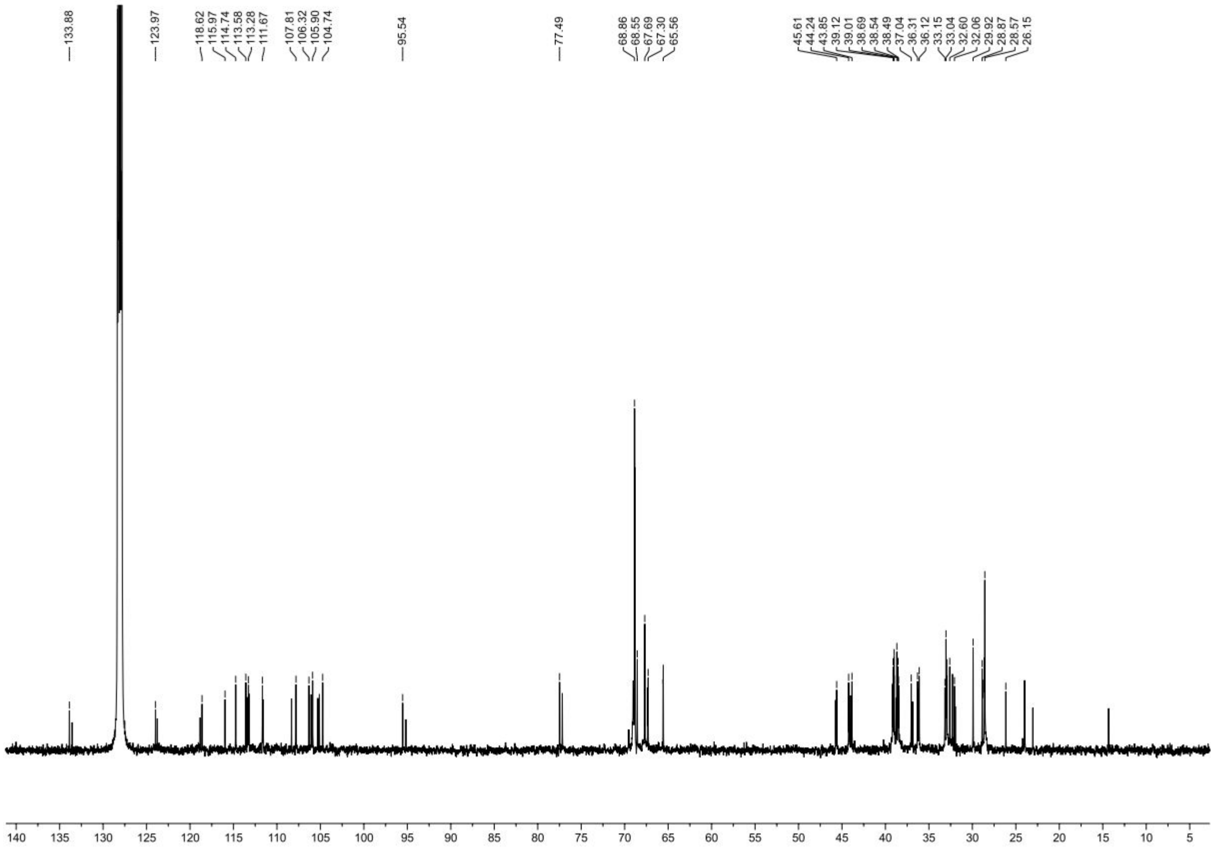

Figure S10: ^{13}C NMR spectrum (125 MHz, C_6D_6, 305 K) of **Ti2b**.

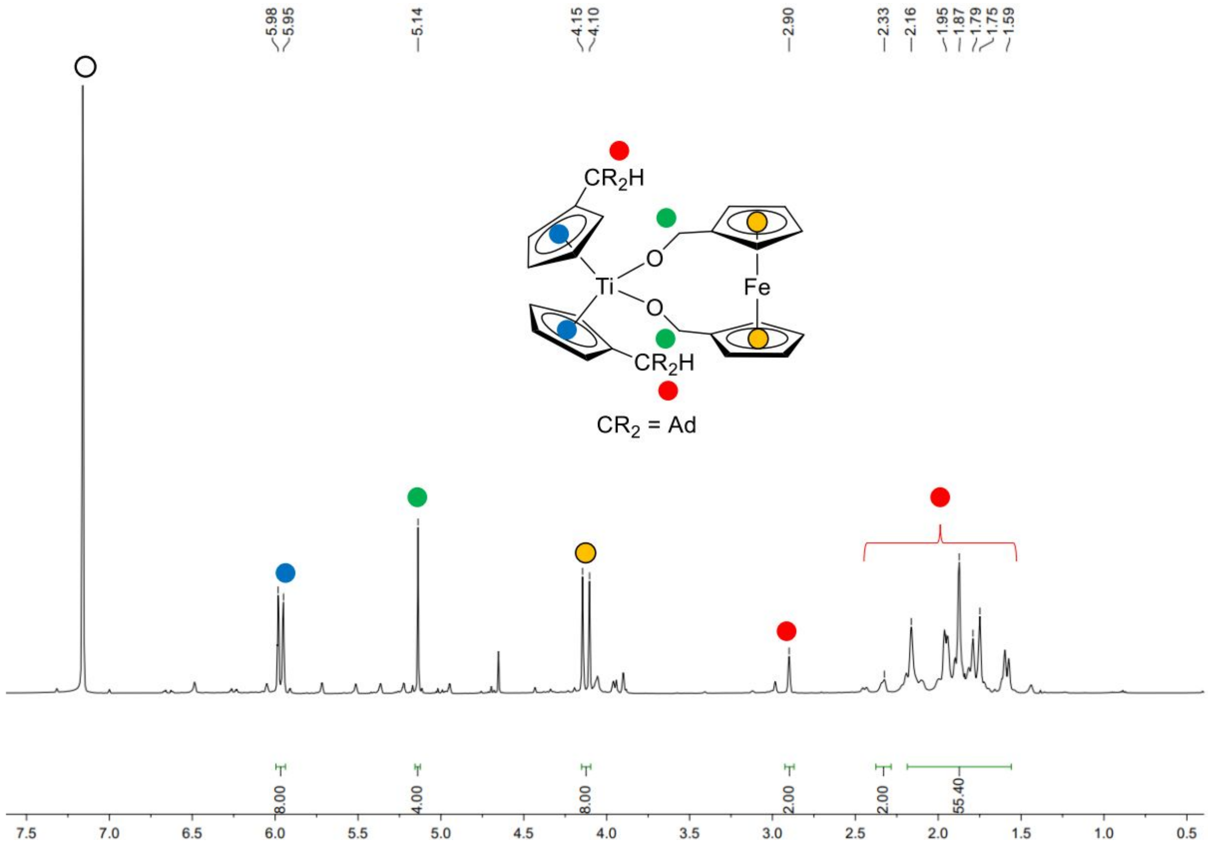

Figure S11: ^{1}H NMR spectrum (500 MHz, C_6D_6, 305 K) of **Ti3a**. Product signals are given in colours (^{o} = C_6D_5H).

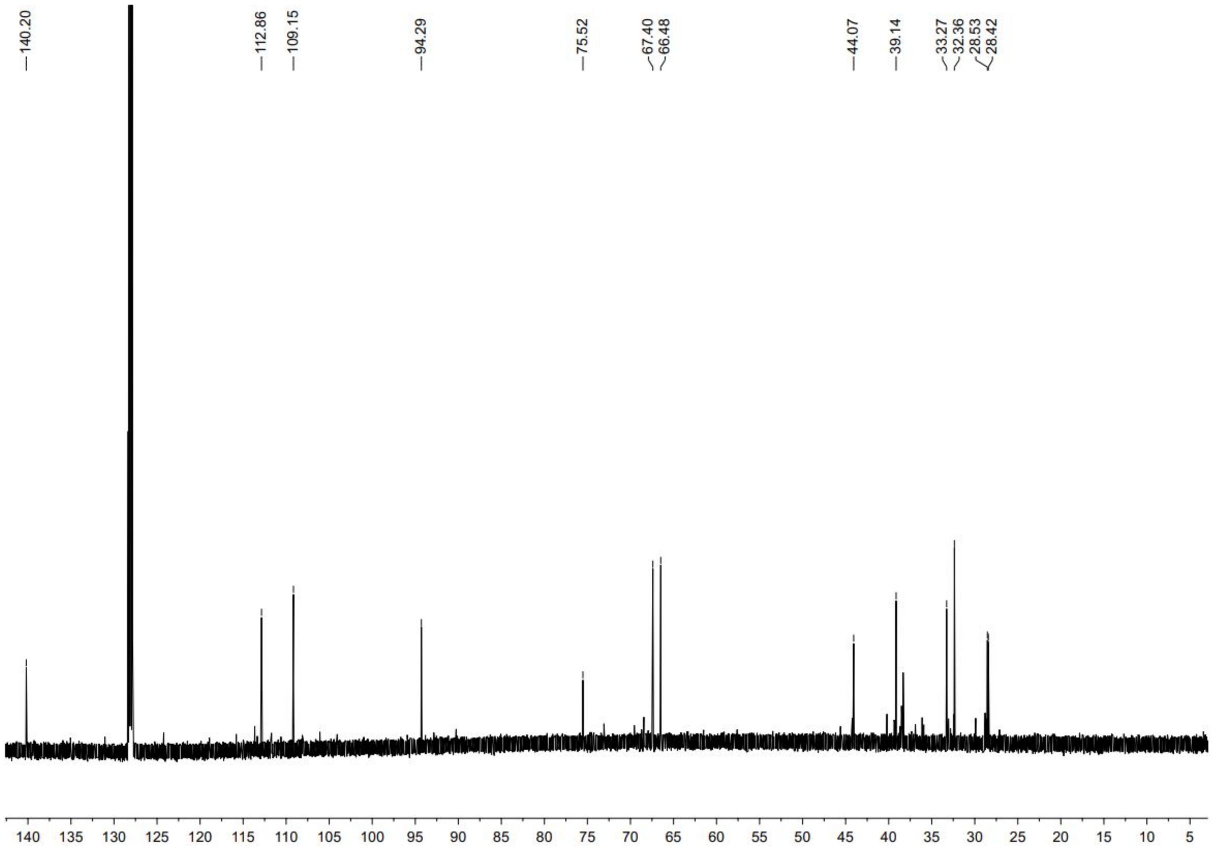

Figure S12: ^{13}C NMR spectrum (125 MHz, C_6D_6, 305 K) of **Ti3a**.

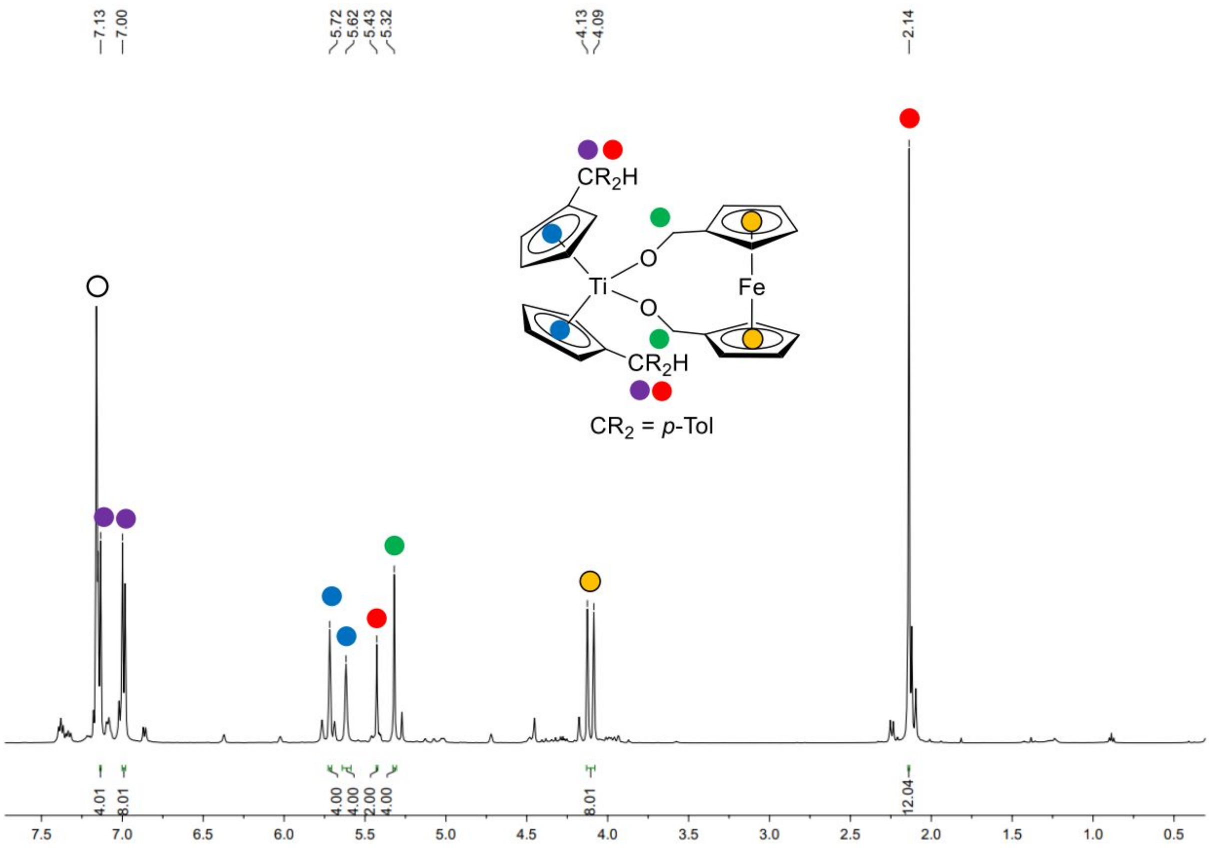

Figure S13: ^{1}H NMR spectrum (500 MHz, C_6D_6, 305 K) of **Ti3b**. Product signals are given in colours (^{o} = C_6D_5H).

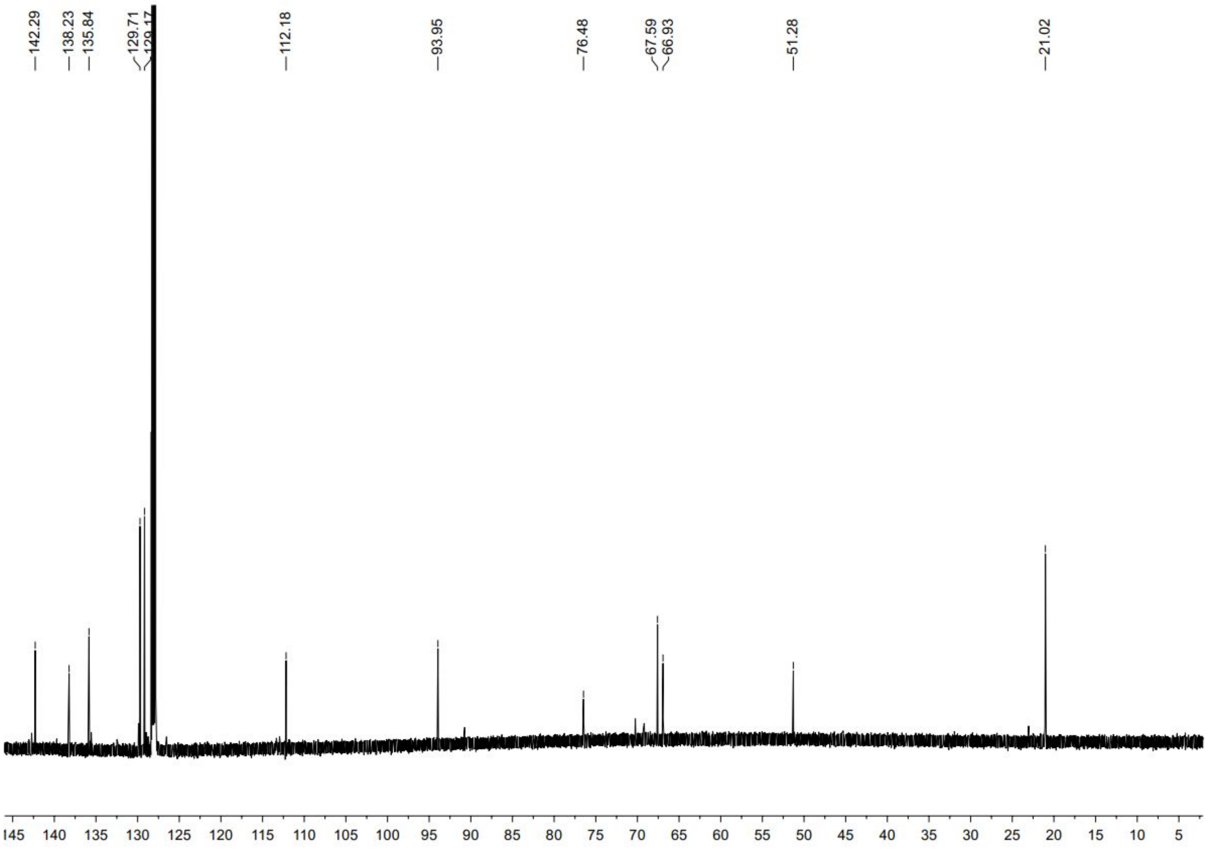

Figure S14: ^{13}C NMR spectrum (125 MHz, C_6D_6, 305 K) of **Ti3b**.

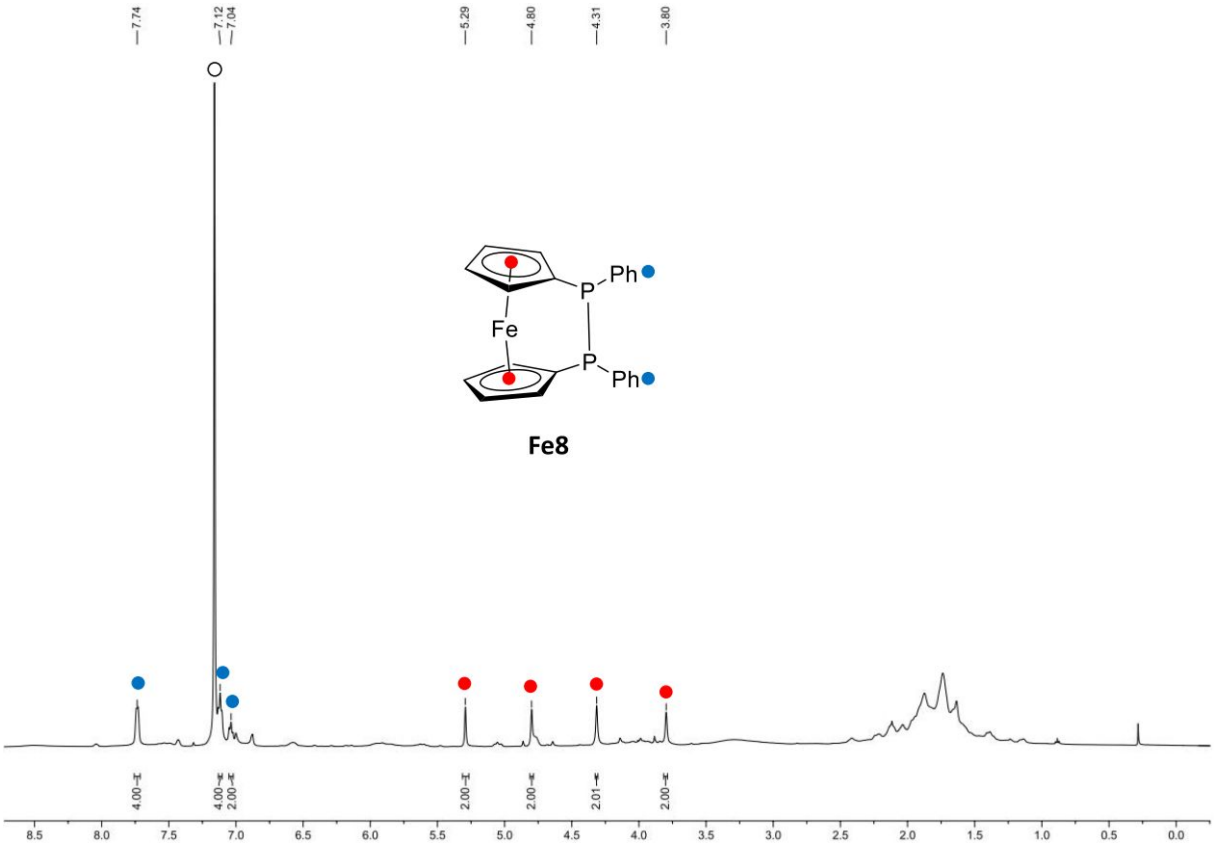

Figure S15: ^{1}H NMR spectrum (500 MHz, C_6D_6, 305 K) of the reaction of **I** with **Fe7**. Product signals of **Fe8** are given in colours (^{o} = C_6D_5H).

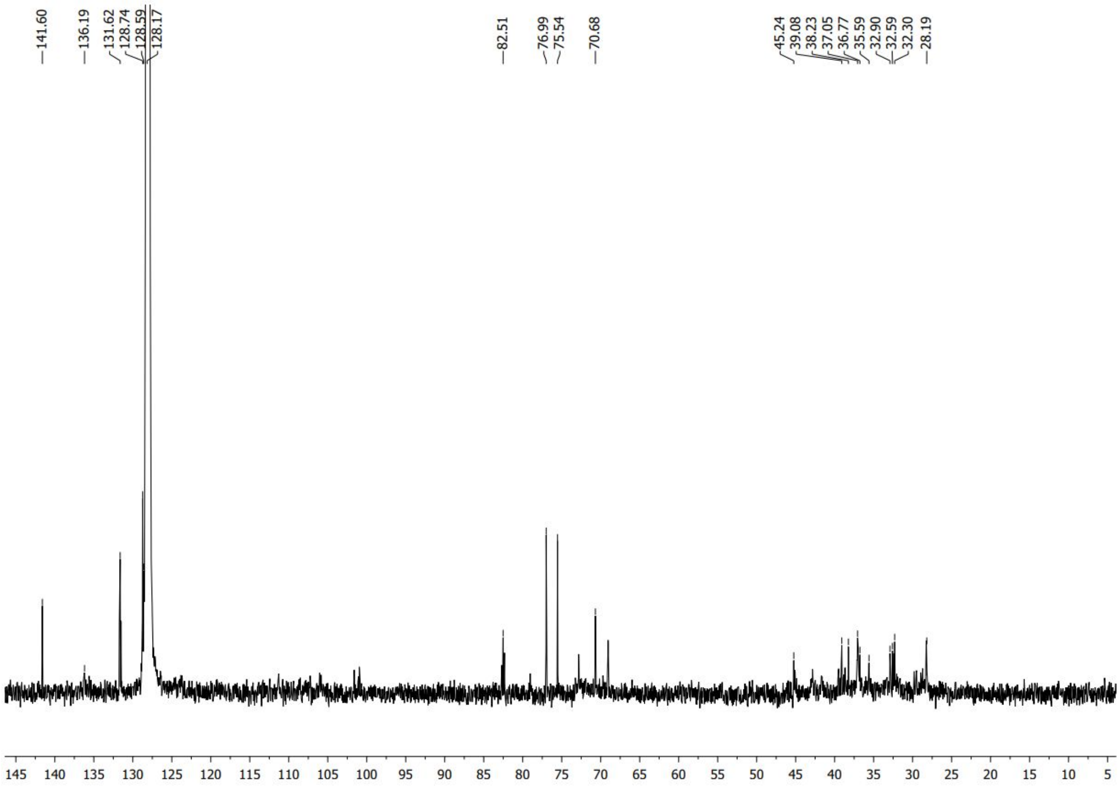

Figure S16: $^{13}C\{^1H\}$ NMR spectrum (125 MHz, C_6D_6, 305 K) of product mixture of the reaction of **I** with **Fe7**.

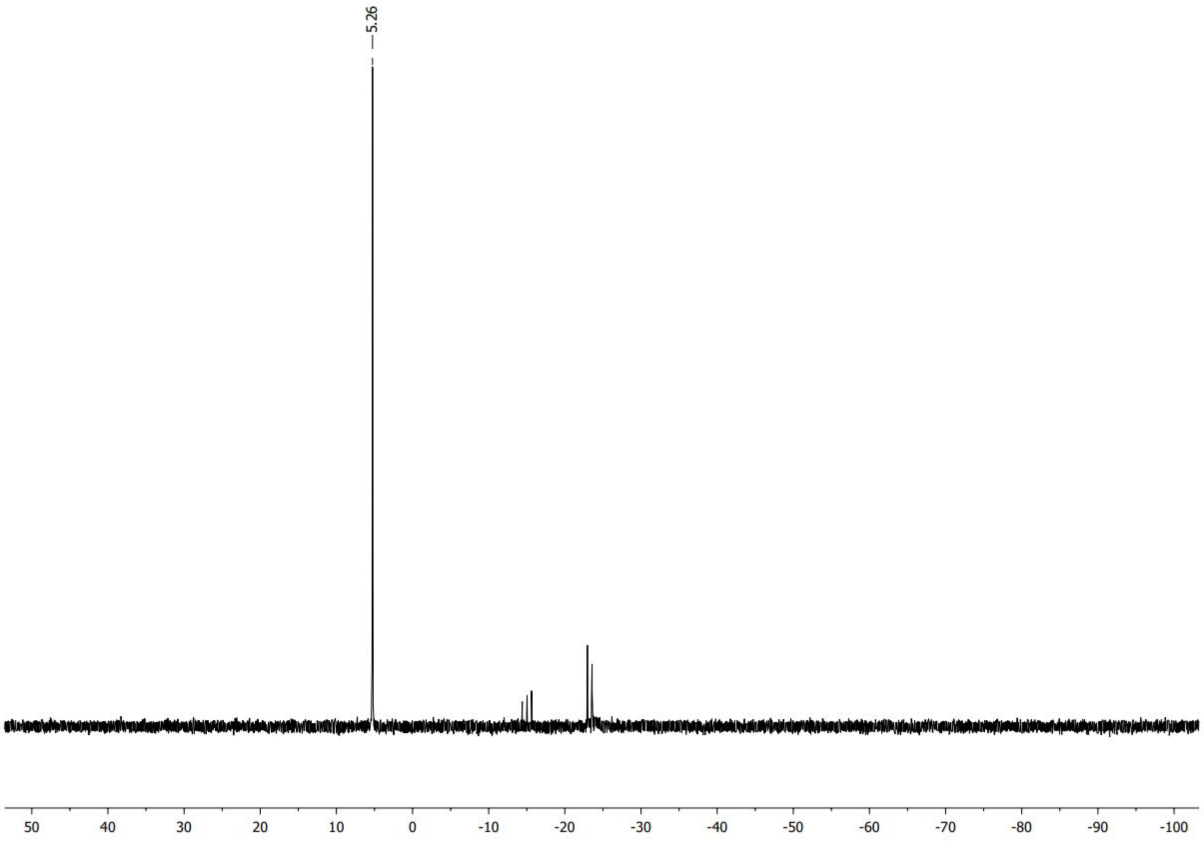

Figure S17: $^{31}P\{^{1}H\}$ NMR spectrum (202 MHz, C_6D_6, 305 K) of **Fe8**.

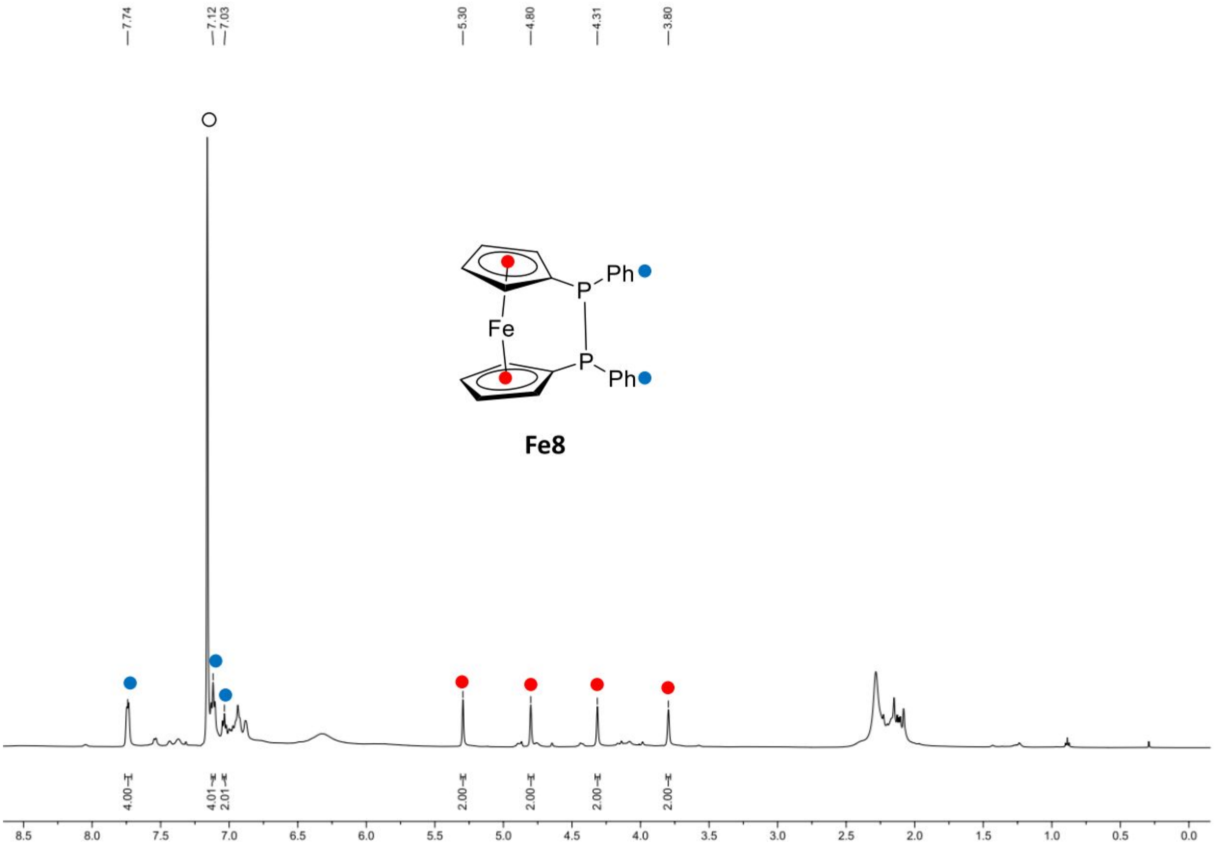

Figure S18: ^{1}H NMR spectrum (500 MHz, C_6D_6, 305 K) of the reaction of **II** with **Fe7**. Product signals of **Fe8** are given in colours (^{o} = C_6D_5H).

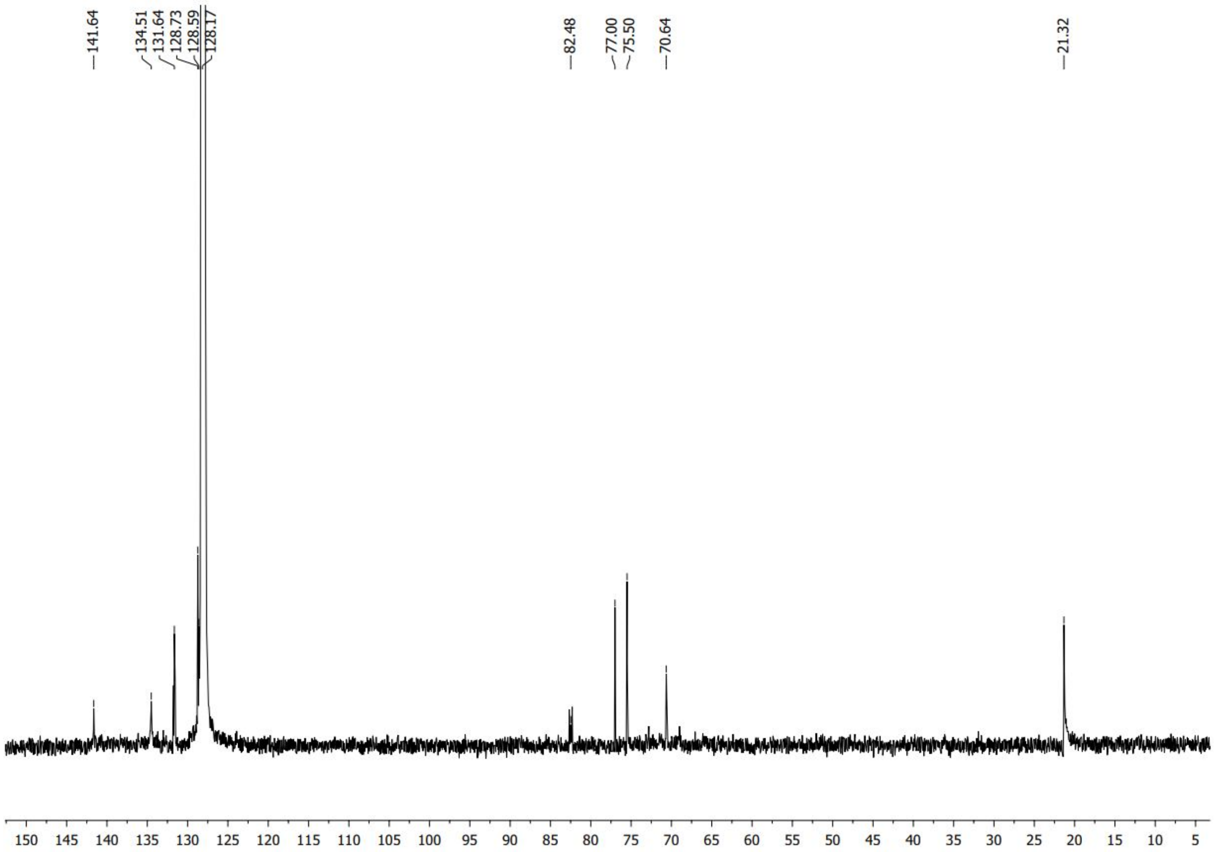

Figure S19: $^{13}C\{^{1}H\}$ NMR spectrum (125 MHz, C_6D_6, 305 K) of product mixture of the reaction of **II** with **Fe7**.

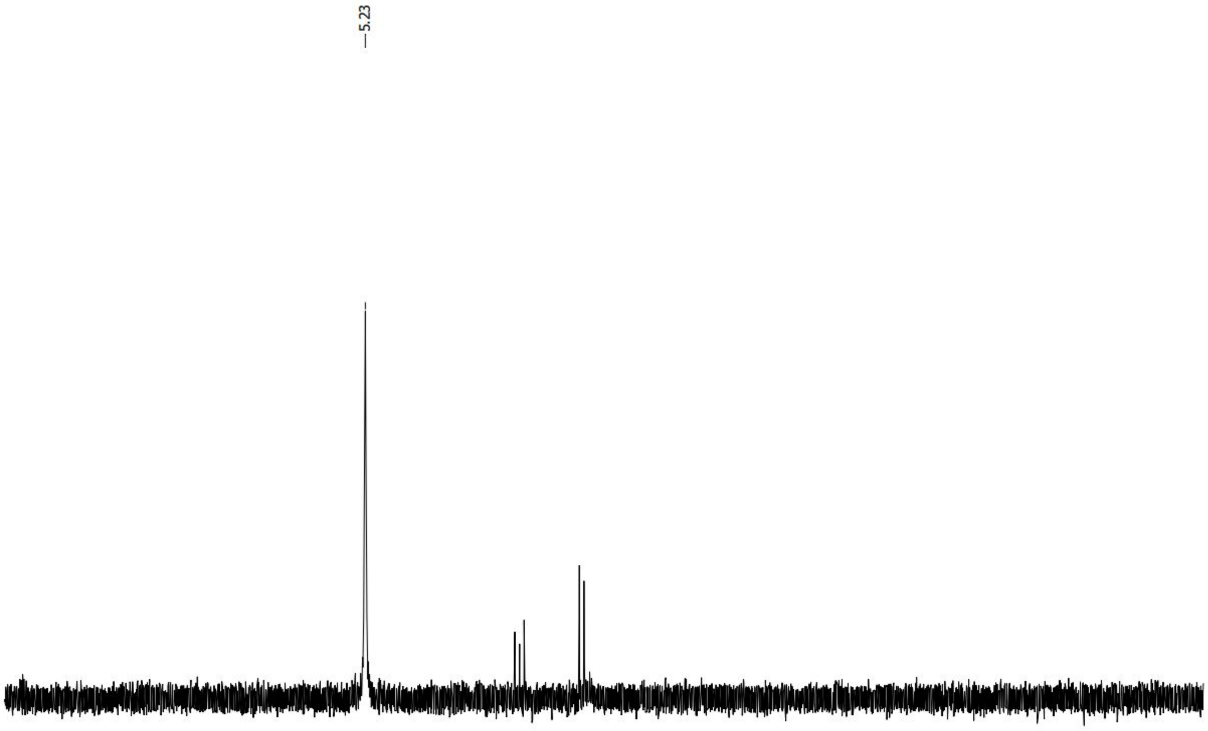

Figure S20: $^{31}P\{^{1}H\}$ NMR spectrum (202 MHz, C_6D_6, 305 K) of **Fe8**.

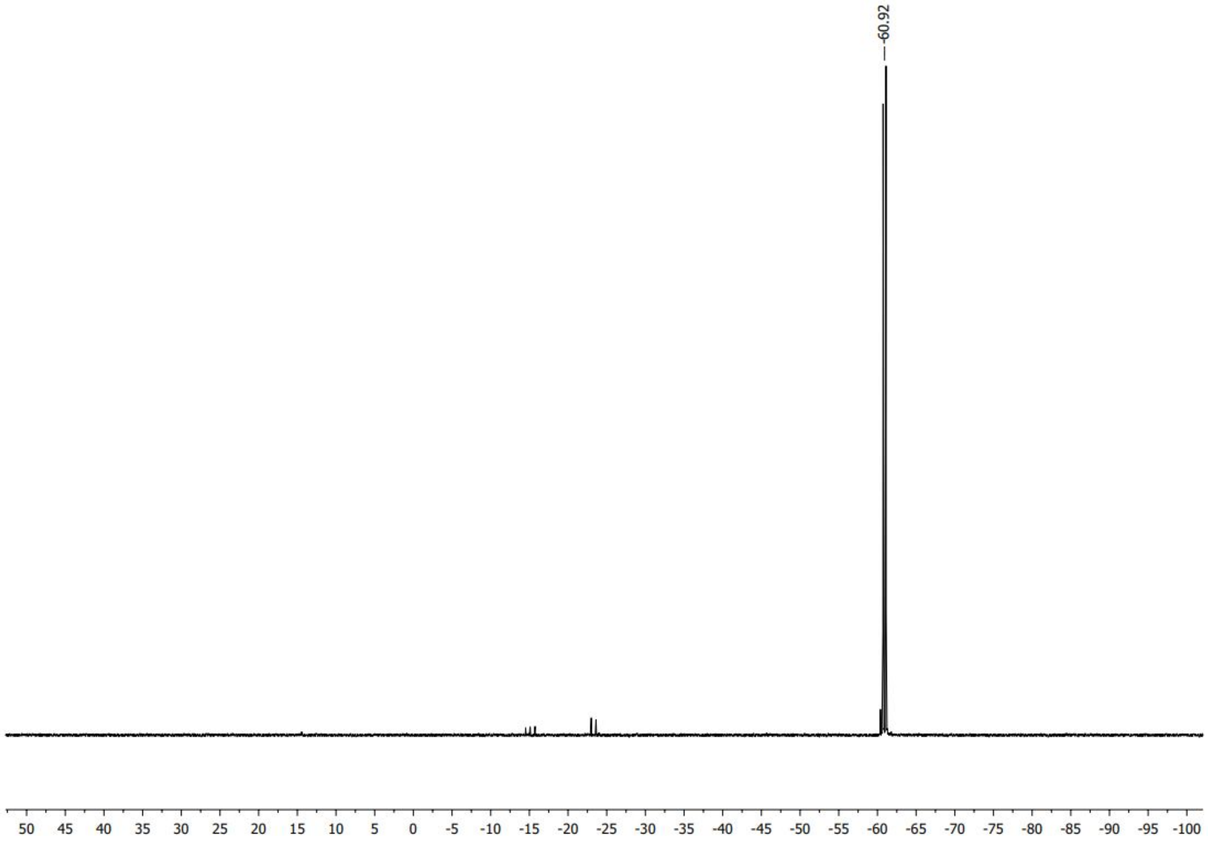

Figure S21: $^{31}P\{^{1}H\}$ NMR spectrum (202 MHz, C_6D_6, 305 K) of **Fe7**.

Crystallographic data

Single crystal X-ray data were measured on a Bruker AXS D8 Venture diffractometer (multilayer optics, Mo-Kα and Cu-Kα radiation with λ = 0.71073 Å and 1.54178 Å respectively, Kappa 4-circle goniometer, Photon III C14 CPAD detector). All crystals were measured at a temperature of 100 K. Absorption corrections using equivalent reflections were performed with the program SADABS.[1] The crystals of **Ti1a** and **AgP** were non-merohedral twins, and the absorption corrections were performed with the program TWINABS.[2] All structures were solved with the program SHELXS[3] and refined with SHELXL[4] using the OLEX2[5] GUI.

All non-H atoms were refined using anisotropic atomic displacement parameters (ADPs). H atoms bonded to C were located in the difference Fourier maps and placed on idealized geometric positions with idealized atomic displacement parameters using the riding model. H atoms bonded to N, O and P were refined freely.

Some of the datasets presented here are troublesome. In particular, the twinned crystals yielded only poor datasets. For **Ti1a** only the heavy atoms Fe and Ti were refined using anisotropic atomic displacement parameters (ADPs). The C and O atoms were refined using isotropic APDs to avoid non positive definites. Inspection of the $F_o - F_c$ plot suggests further twinning beyond the two domain non-merohedral twinning used for processing the current dataset, but no additional reasonable domain could be found. The crystal of **AgP**, in addition to being twinned, decomposed during the data collection, probably due to radiation damage using the high intensity Cu source, which resulted in an incomplete dataset and poor *R* values. The crystal of **Fe8** also was not a single crystal but treating the data as a (non-merohedral) twin did not improve the refinement results. Due to the poor data quality of the structures above we abstained from a detailed discussion of bond length, etc.. The structures are presented here nonetheless in support of the other analytical data (NMR, etc.).

Data for the otherwise good crystal **Ti2b** were only measured to low resolution (0.98 Å). The structure of **Ti4** exhibits a comparatively large residual density peak (3.5 $e \cdot Å^{-1}$ max. near Fe1), pointing to an unresolved disorder of the ferrocene ligand amounting to approximately 3% when the residual density peak is refined as Fe.

The crystallographic data can be obtained free of charge from https://www.ccdc.cam.ac.uk/structures/ quoting the CCDC numbers 2338540-2338546.

Table S1: Crystallographic data of **Ti1a**, **Ti2a**, **Ti2b**, **Ti3b**, **Fe8**.

	**Ti1a**	**Ti2a**	**Ti2b**	**Ti3b**	**Fe8**
CCDC	2338542	2338543	2338544	2338545	2338541
Lab-ID	JUKL013	JUKL024	KESC96	KESC249	KESC231
empirical formula	$C_{41}H_{46}FeOTi$	$C_{41}H_{48}FeOTi$	$C_{42}H_{50}FeOTi$	$C_{52}H_{50}FeO_2Ti$	$C_{22}H_{18}FeP_2$
Fw	658.53	660.54	674.57	810.67	400.15
Colour	orange	red	brown	yellow	orange
Habit	plate	needle	plate	block	block
cryst. dimens. mm	0.14 x 0.13 x 0.01	0.10 x 0.01 x 0.01	0.09 x 0.07 x 0.02	0.14 x 0.13 x 0.06	0.06 x 0.05 x 0.03
cryst. system	triclinic	monoclinic	triclinic	monoclinic	monoclinic
space group	P-1	$P2_1/c$	P-1	C2/c	$P2_1/n$
a, Å	10.2052(9)	19.7613(6)	12.2961(5)	23.2308(11)	11.7077(4)
b, Å	14.0753(11)	20.0088(6)	12.7995(5)	15.0257(7)	7.2969(3)
c, Å	21.6199(19)	7.8408(2)	21.3801(9)	11.5826(5)	40.9612(15)
α, deg	95.506(3)	90	96.559(2)	90	90
β, deg	99.914(4)	97.2996(15)	91.847(2)	96.6623(18)	91.2663(17)
γ, deg	90.004(3)	90	104.563(2)	90	90
V, $Å^3$	3044.6(4)	3075.12(15)	3229.0(2)	4015.7(3)	3498.5(2)
Z	4	4	4	4	8
$D_{calc.}$, g cm^{-3}	1.437	1.427	1.388	1.341	1.519
μ, mm^{-1}	0.773	6.233	5.948	0.602	8.632
T, K	100(2)	100(2)	100(2)	100(2)	100(2)
λ, Å	0.71073	1.54178	1.54178	0.71073	1.54178
θ range, deg	1.454 – 30.034	2.254 – 74.487	2.084 – 51.746	1.617 – 40.249	2.158 – 74.489
reflections collected	35724	71520	61785	181982	61861
Indep. Reflecions R(int)	35724 ?	6279 0.0538	7076 0.0343	12644 0.0378	7099 0.0634
Observed reflections (I > 2(I))	29644	5660	6707	11565	6806
Absorption correction	semi-empirical	semi-empirical	semi-empirical	semi-empirical	semi-empirical
max, min transm.	1.0000, 0.8288	1.0000, 0.8288	1.0000, 0.7636	1.0000, 0.9178	1.0000, 0.7911
final R indices [I>2σ(I)]	R1 = 0.0921, wR2 = 0.2232	R1 = 0.0293, wR2 = 0.0730	R1 = 0.0259, wR2 = 0.0684	R1 = 0.0313, wR2 = 0.0890	R1 = 0.0825, wR2 = 0.2276
R indices (all data)	R1 = 0.1117, wR2 = 0.2343	R1 = 0.0349, wR2 = 0.0765	R1 = 0.0277, wR2 = 0.0696	R1 = 0.0347, wR2 = 0.0911	R1 = 0.0842, wR2 = 0.2285
GOF on F^2	1.163	1.020	1.027	1.085	1.168
largest diff peak / hole (e.Å- ³)	0.756 / -0.407	0.465 / -0.289	0.233 / -0.293	0.688 / -0.730	1.594 / -0.771

Table S2: Crystallographic data of **Ti4**, **AgP**.

	**Ti4**	**AgP**
CCDC	2338546	2338540
Lab-ID	KESC232	KESC237
empirical formula	$C_{32}H_{29}FeP_2Ti$	$C_{55.5}H_{52}AgF_3Fe_2O_3P_4S$
Fw	579.24	1199.48
Colour	brown	green yellow
Habit	plate	plate
cryst. dimens. mm	0.10 x 0.08 x 0.05	0.05 x 0.05 x 0.01
cryst. system	triclinic	triclinic
space group	P-1	P-1
a, Å	9.7842(6)	10.5563(7)
b, Å	10.9775(6)	13.4287(9)
c, Å	13.5173(8)	20.7698(14)
α, deg	107.556(2)	101.565(5)
β, deg	92.610(2)	100.636(5)
γ, deg	110.933(2)	107.371(4)
V, $Å^3$	1273.55(13)	2657.2(3)
Z	2	2
$D_{calc.}$, g cm^{-3}	1.511	1.499
μ, mm^{-1}	1.030	9.192
T, K	100(2)	100(2)
λ, Å	0.71073	1.54178
θ range, deg	1.604 – 34.969	2.249 – 66.587
reflections collected	123601	21650
Indep. Reflecions<br>R(int)	11183<br>0.0508	21650<br>?
Observed reflections (I > 2(I))	9875	12214
Absorption correction	semi-empirical	semi-empirical
max, min transm.	1.0000, 0.9107	1.000000, 0.665310
final R indices [I>2σ(I)]	R1 = 0.0485, wR2 = 0.1131	R1 = 0.0948, wR2 = 0.2158
R indices (all data)	R1 = 0.0571, wR2 = 0.1176	R1 = 0.1679, wR2 = 0.2562
GOF on F^2	1.122	1.059
largest diff peak / hole (e.Å- ³)	3.502 / -0.729	1.279 / -0.887

Cyclic voltammograms of precursors

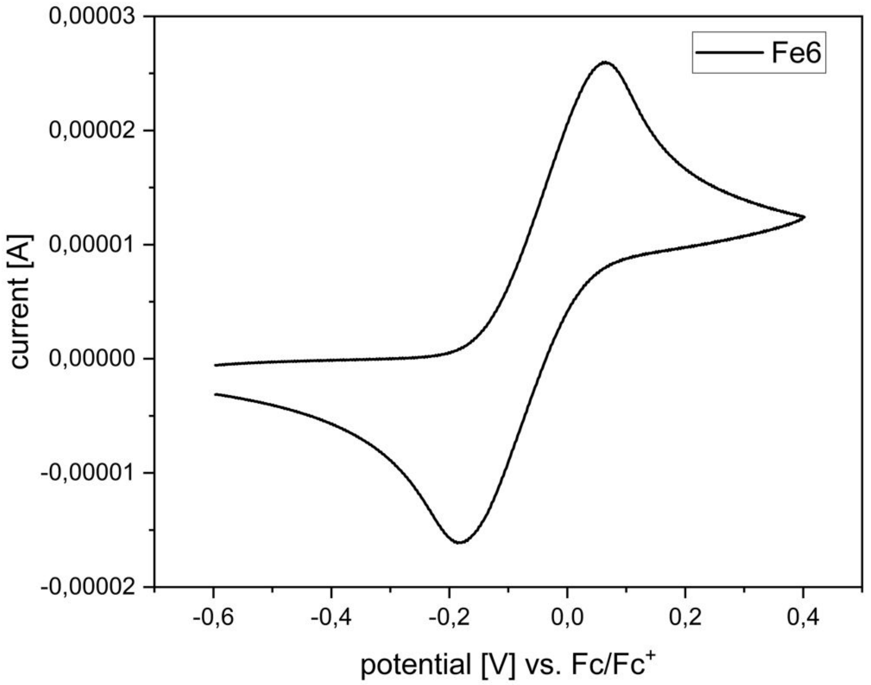

Figure S22. Cyclic voltammogram of **Fe6**. Measurements were carried out in THF at room temperature under argon atmosphere (supporting electrolyte [*n*-Bu_4N]PF_6, 0.1 M, scan rate 0.1 V/s). Potentials were referenced to the ferrocene/ferrocene$^+$ redox couple with $E_{1/2}$ = 0.00 V.

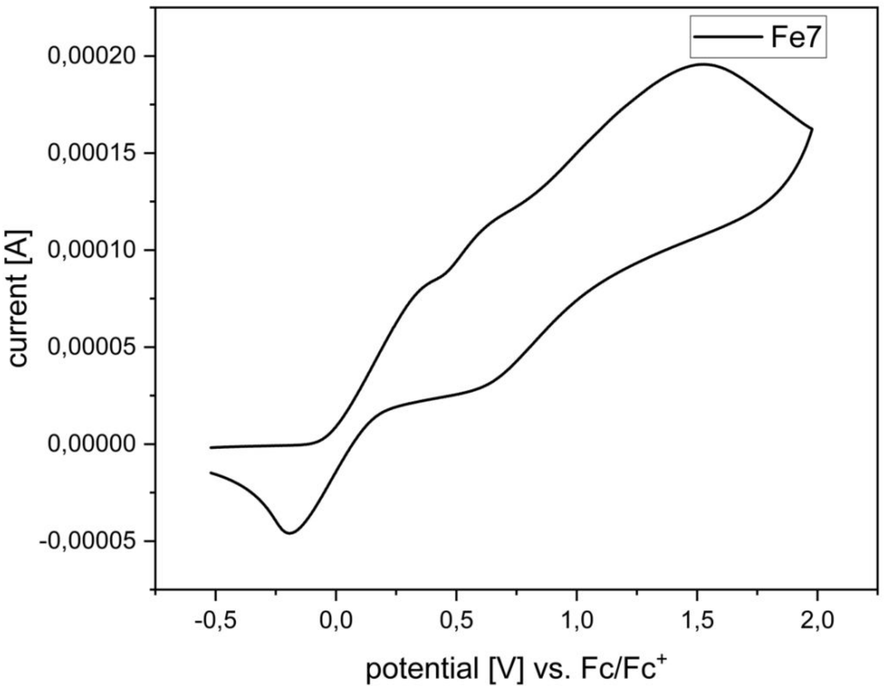

Figure S23. Cyclic voltammogram of **Fe7**. Measurements were carried out in THF at room temperature under argon atmosphere (supporting electrolyte [*n*-Bu_4N]PF_6, 0.1 M, scan rate 0.1 V/s). Potentials were referenced to the ferrocene/ferrocene$^+$ redox couple with $E_{1/2}$ = 0.00 V.

References

[1] L. Krause, R. Herbst-Irmer, G. M. Sheldrick, D. Stalke, Journal of Applied Crystallography 2015, 48, 3-10.

[2] G. M. Sheldrick, TWINABS 2012/1, Bruker, Madison, Wisconsin, USA, 2012.

[3] G. M. Sheldrick, Acta Crystallographica Section A 2008, 64, 112-122.

[4] G. M. Sheldrick, Acta Crystallographica Section C 2015, 71, 3-8.

[5] O. V. Dolomanov, L. J. Bourhis, R. J. Gildea, J. A. K. Howard, H. Puschmann, Journal of Applied Crystallography 2009, 42, 339-341.
